# Identifying Single Copy Orthologs in Metazoa

**DOI:** 10.1371/journal.pcbi.1002269

**Published:** 2011-12-01

**Authors:** Christopher J. Creevey, Jean Muller, Tobias Doerks, Julie D. Thompson, Detlev Arendt, Peer Bork

**Affiliations:** 1 Teagasc, Animal & Grassland Research and Innovation Centre, Grange, Dunsany, County Meath, Ireland; 2 European Molecular Biology Laboratory, Heidelberg, Germany; 3 Laboratoire de Diagnostic Génétique, CHU Strasbourg Nouvel Hôpital Civil, Strasbourg, France; 4 IGBMC (Institut de Génétique et de Biologie Moléculaire et Cellulaire), CNRS/INSERM/Université de Strasbourg, Illkirch Cedex, France; 5 Max-Delbrück-Centre for Molecular Medicine, Berlin, Germany; Cornell University, United States of America

## Abstract

The identification of single copy (1-to-1) orthologs in any group of organisms is important for functional classification and phylogenetic studies. The Metazoa are no exception, but only recently has there been a wide-enough distribution of taxa with sufficiently high quality sequenced genomes to gain confidence in the wide-spread single copy status of a gene.

Here, we present a phylogenetic approach for identifying overlooked single copy orthologs from multigene families and apply it to the Metazoa. Using 18 sequenced metazoan genomes of high quality we identified a robust set of 1,126 orthologous groups that have been retained in single copy since the last common ancestor of Metazoa. We found that the use of the phylogenetic procedure increased the number of single copy orthologs found by over a third more than standard taxon-count approaches. The orthologs represented a wide range of functional categories, expression profiles and levels of divergence.

To demonstrate the value of our set of single copy orthologs, we used them to assess the completeness of 24 currently published metazoan genomes and 62 EST datasets. We found that the annotated genes in published genomes vary in coverage from 79% (*Ciona intestinalis*) to 99.8% (human) with an average of 92%, suggesting a value for the underlying error rate in genome annotation, and a strategy for identifying single copy orthologs in larger datasets. In contrast, the vast majority of EST datasets with no corresponding genome sequence available are largely under-sampled and probably do not accurately represent the actual genomic complement of the organisms from which they are derived.

## Author Summary

The correct identification of single copy (1-to-1) orthologs is crucial for functional classification of genes and for phylogenetic studies of groups of organisms, including the Metazoa. Nevertheless, despite the recent increase in the number of genomes and short sequence read datasets (e.g. ESTs) from the Metazoa, we know little about their completeness and how useful they may be for phylogenetic studies. Here we describe a novel approach for the identification of single copy gene families at any hierarchical level and demonstrate its effectiveness by identifying a set of over one thousand gene families that have been in single copy since the last common ancestor of the Metazoa. By comparing our orthologs to those predicted by other datasets we show that our procedure identifies a significantly larger set of single copy orthologs in the Metazoa. We then use this dataset to assess 24 metazoan genomes and 61 metazoan EST datasets for their completeness. We thus identify the underlying error rate in genome annotation and suggest a mechanism for assessing the quality of genomes and EST datasets in terms of their suitability for phylogenetic studies.

## Introduction

Not long after the release of the first bacterial genome sequence [1], large-scale identification of gene families from multiple organisms became feasible [2]–[5] and allowed them to be classified into groups according to their homologous relationships [6]. These classifications now represent a widely used resource for various applications [7].

For many applications, it is critical to describe these homologous relationships in more detail by differentiating orthologs from paralogs [6]. Orthologs are genes that diverged through a speciation event, as opposed to paralogous genes, which diverged after a duplication event [8]. Unfortunately, in practice, the identification and classification of orthologous genes remains very difficult and relies on operational definitions [9]. Several conceptually different approaches have been developed that aim to establish these relationships between genes from different genomes [10]–[13]. The methods are generally based on sequence alignments between pairs of sequenced genomes where reciprocal best alignments are used to define orthologs [13] and several online databases now exist that provide pre-calculated sets at different taxonomic levels [14]–[17]. Since defining a clear 1-to-1 relationship between two genes is sometimes complex, operational orthologous groups have been introduced [7] that allow difficult cases to be resolved, although these groups depend on the genomes and taxonomic levels used to derive the respective gene sets [6]. This is illustrated nicely with an example from the eggNOG database version 1 (evolutionary genealogy of genes: Non-supervised Orthologous Groups) [14] which groups genes into families at different taxonomic levels balancing phylogenetic coverage and resolution. At the metazoan level in eggNOG (i.e. metazoan Non-supervised Orthologous Groups or meNOG), all myosins form a single orthologous group (meNOG06059) as the differing body plans across the animals do not allow a more specific classification. However, when considering the mammalian level (i.e. mammalian Non-supervised Orthologous Groups or maNOG), the myosins are divided into 5 gene families with separate annotated functions (maNOG16585 - cardiac muscle; maNOG08909 - skeletal muscle protein; maNOG04095 - motor protein; maNOG16587 - striated muscle contraction and maNOG17387 - myosin-1) [14]. At any taxonomic level, the identification of single copy (or 1-to-1) orthologs is important for phylogenetic measures while, 1-to-many and many-to-many relationships of genes between sequenced genomes reveal functional differences [18], [19].

The definition of genes in a pair of species as single copy orthologs implies that they have kept this status since the species last shared a common ancestor [20] (although it does include rare complex scenarios, such as the differential loss of paralogs after whole genome duplication [21] or orthologous gene displacement [22]). The single copy status of such genes makes them very useful for a variety of comparative genomic approaches such as large-scale phylogenetic reconstructions [23]–[26], and assessments of completeness of sequenced genomes [27]–[29]. Regardless of the methods used to create the gene sets (or families), single copy gene families are identified by counting the number of representatives of each species in the family in question. Due to problems of genome incompleteness or misannotation, a tolerance, e.g. of plus or minus one copy from a single organism, has been shown to increase prediction sensitivity [30]. Nevertheless, this arbitrary value is insensitive to the number of genomes in the study, and while some attempts at estimating the underlying stochastic error in low coverage genomes have been made [31] no wide-scale adjustments can be done. Furthermore, ortholog datasets constructed using different methods can differ greatly, making comparisons between different sets of single copy orthologs meaningless [32], [33]. Finally, standard taxon-count methods discard many multigene families containing subsets of single copy orthologs. As a result, a potentially large proportion of phylogenetically useful genes are excluded from subsequent analyses.

To address these issues, we have developed a phylogenetic approach for identifying overlooked single copy orthologs within multigene families and applied it to a minimal set of (18) high quality metazoan genomes spanning multiple metazoan phyla. We identified a set of 1,126 single copy orthologs representing a wide range of functional classes, expression profiles and evolutionary rates. These ortholog sets were then used to assess 24 metazoan genomes and 61 publicly available sets of ESTs from a wide selection of metazoan groups for their completeness.

## Results/Discussion

### Identifying single copy metazoan orthologs

We assembled all the gene families from a minimal set of 18 metazoan genomes using eggNOG version 1 [14]. The genomes were chosen on the basis that they have been in the public domain long enough to have been improved and refined (Supplemental Table S1). The choice of genomes is critical for our purposes, since we need to balance the quality of the dataset used to ensure confidence in our results and a wide enough distribution of distinct lineages to enable us to assess the true status of metazoan single copy orthologs.

Firstly, single-copy orthologs were identified from the gene families where one copy from each of the 18 metazoan genomes was present. A loss or duplication event in a single genome per family was permitted since (i) many published genomes are not complete, (ii) gene predictions are not perfect and (iii) in some genomes, pseudogenes are not annotated as such, thus appearing as artificial duplications. This resulted in 219 genes with exactly one ortholog in each genome examined, 125 genes that were duplicated in only a single genome and 478 genes that were lost in only a single genome, with an average coverage of 92% per genome. Given that both duplication and loss events are likely to occur at rates determined by the molecular clock [18], the much higher number of losses seems to indicate a considerable incompleteness of the published genomes (see Supplemental Table S2 for more details). This hypothesis provides a strategy for estimating the underlying stochastic error rate in genome annotation in other datasets.

Secondly, a gene-tree reconciliation approach [34] was used to identify sub-trees of multigene families where the sub-tree contains only single copy orthologs and no duplications or losses have been observed since the last common ancestor of Metazoa. These sub-trees will be referred to as ‘single copy sub-trees’ hereafter. This procedure necessitated the construction of robust gene-trees for over 20,000 multigene families, as well as a “species” tree from 40 universally distributed single copy gene families (Figure 1.1) [35]. The species tree was then used as a guide to construct a reconciled tree for each multigene family, where the history of the gene tree was embedded in the species tree. We then calculated the number of duplications and losses that are required to explain the topology of the gene tree, given the species tree. As this is dependent upon the root chosen for the gene-tree, all possible rootings were assessed for each gene tree, and the one that minimized the number of duplications and losses was considered to be the most parsimonious (Figure 2) [36].

**Figure 1:**
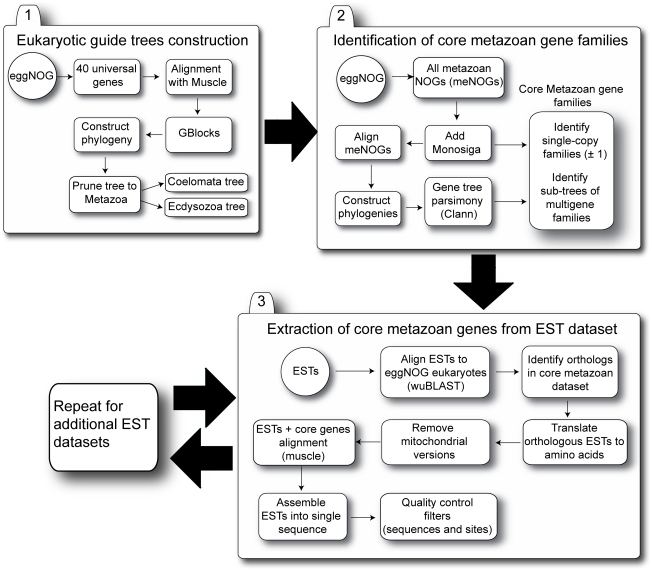
Project workflow. The analysis workflow is divided into 3 major steps. The first step (Eukaryotic guide tree construction) aims at constructing the guide tree used to infer duplication and loss events. The second step (Identification of core metazoan gene families) is the core of our method, i.e. the identification within the eggNOG database of the single copy genes. The last step concerns the extraction of the single copy genes from the EST datasets.

**Figure 2:**
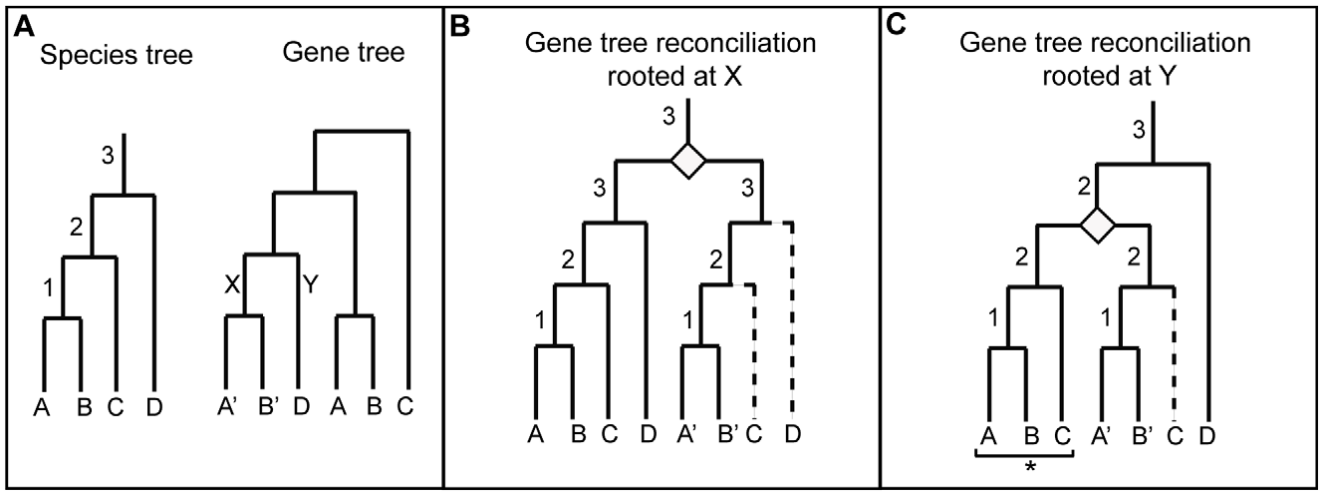
Gene tree reconciliation process. Reconciling a gene tree with a (guide) species tree. A) Given the species tree on the left, we need to estimate the most parsimonious number of duplications and losses that explain the topology and distribution of the gene tree (on the right). In order to assess correctly the number of duplications and losses, we need to find the best rooting of the gene tree. To this end, the gene tree is rooted at every possible position, and for each rooting, the most parsimonious number of duplications and losses is calculated. The rooting that requires the fewest number of steps (duplications and losses) is considered the most parsimonious rooting of the gene tree. For example: the reconciliations for two possible rootings are shown: positions X and Y in panes B) and C). The positions of duplication events are indicated with a diamond, losses are indicated with a dashed line. B) Rooting the gene tree at position X in B) requires duplication and two losses, while rooting at position Y in C) requires 1 duplication and 1 loss. Of the two rootings, position Y is the most parsimonious. The numbers on the internal branches indicate the internal branch of the species tree in A)that they are mapped to. If we were trying to identify single copy genes at the hierarchical level of internal branch 2 on the species tree, then the sub-tree marked with a * in C) would represent a gene family that has been in single copy since this hierarchical level.

**Figure 3:**
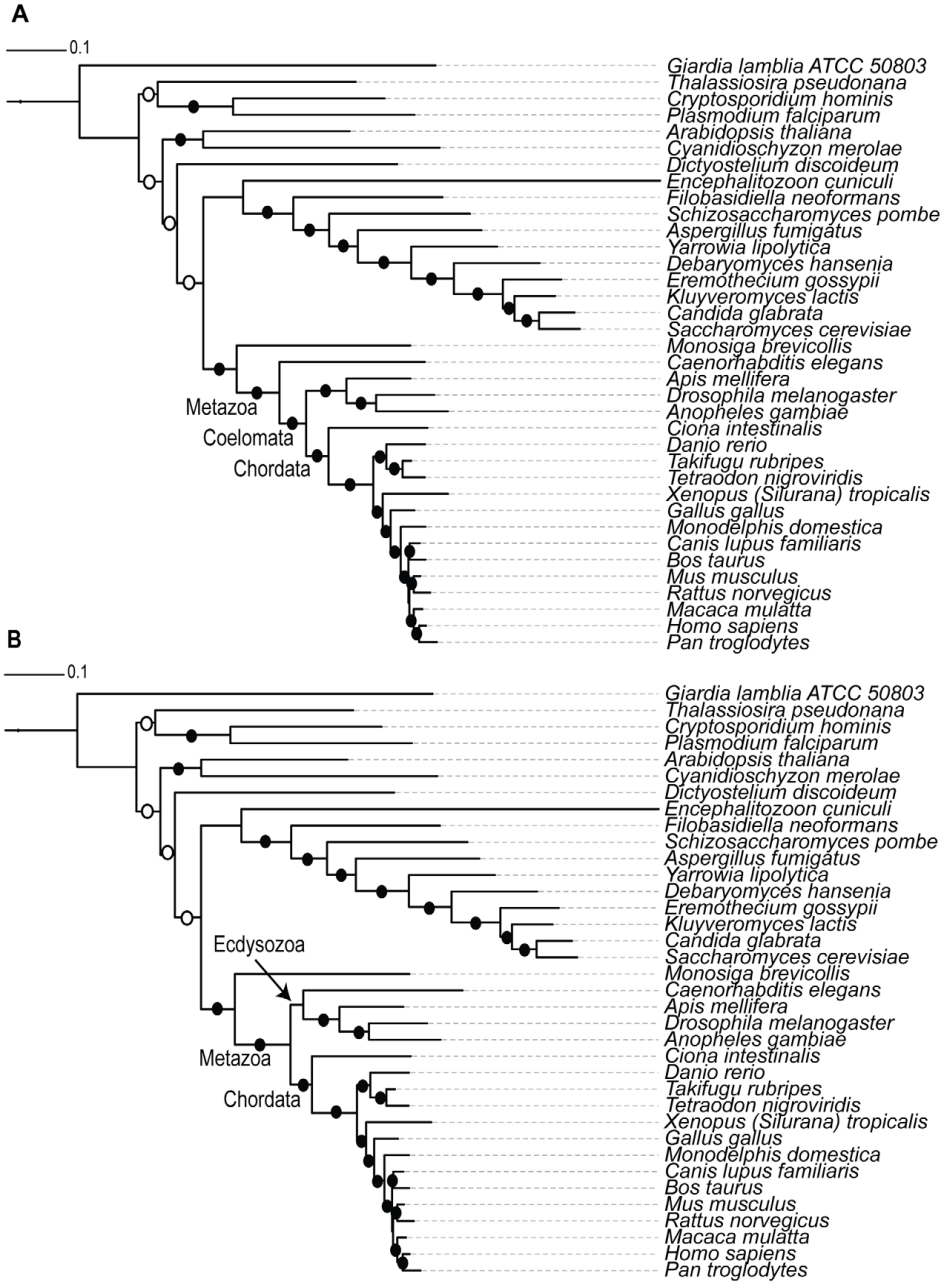
Eukaryotic guide trees used in the analysis. The Eukaryotic guide trees constructed based on a concatenated alignment of the 40 universally distributed genes [35]. A) The phylogeny supporting the Coelomata hypothesis for the evolution of animals. B) The phylogeny supporting the Ecdysozoa hypothesis for the evolution of animals was created by hand from A). Branch lengths represent the evolutionary distances between the taxa based on their amino acid sequences and were estimated using the same alignments of universal genes. Both trees were used in the gene-tree reconciliation step, so as not to bias subsequent analyses towards either hypothesis. Filled circles represent internal branches that received greater than 95% Bootstrap proportion (BP) support. Open circles represent internal branches with greater than 60% BP support.

Two different species trees were applied in the reconciliation procedure: one supporting the Coelomata hypothesis for animal evolution, although this hypothesis is questionable due to potential long-branch attraction and other issues [37], and one supporting the Ecdysozoan hypothesis for animal evolution (Figure 3). The results from both reconciliations were then pooled. For each gene-tree, the most parsimonious reconciliation for the species trees was used to determine whether there were any single copy sub-trees in the corresponding multigene family (allowing for species-specific duplications or losses) (Figure 2). Using this approach, we identified 304 additional single copy Metazoan orthologs, increasing the number of single-copy orthologs by 36%.

By combining the single gene families and the single copy sub-trees in multigene families, we identified a total of 1,126 single copy metazoan orthologs with an average gene length of 552 amino acids (ranging from 72 to 4,762, see Supplemental Figure S1 and Supplemental Table S3 for more details). Interestingly, the distribution of expression profiles for the human genes in these families showed no significant difference from the distribution of expression profiles for 33,675 human gene transcripts (from across 79 different tissue types), indicating that the single copy orthologs are representative of a wide spectrum of expression profiles (Supplemental Figure S2). Similarly, the distribution of sequence conservation in the 1,126 single copy orthologs is similar to that found in the complete spectrum of meNOGs, ranging from low to highly divergent gene families (Supplemental Figure S3).

### Assessing the level of genome misannotation

To test the validity of our assumption that the loss of a gene in a single lineage (genome) may be representative of misannotation, we examined two genomes that represent extremes in genome annotation quality. *Homo sapiens* (version NCBI 36) was the best annotated genome in our dataset and was missing representatives from only 2 single copy gene families. We compared this with *Pan troglodytes* (version 1.0) which was missing representatives from 231 single copy gene families. We searched the latest annotation of the chimpanzee genome (version 2.1) and identified 115 orthologs of these missing genes. In addition, BLAST [38] searches were performed for a random sample of the remaining 116 genes and homologous regions with high identity were found for all of them. Our findings are corroborated by a recent manual comparison of the single copy orthologous regions between the human and chimpanzee genomes which revealed that only 3 human genes did not have corresponding orthologs in the chimpanzee genome [39].

We then carried out the same procedure for the two single copy genes missing in the human genome. Using NCBI BLAST to search the latest human genome database (build 37), we identified significantly conserved homologous regions for both gene families, further supporting our assumption that a loss in a single genome may be representative of annotation errors rather than a genuine loss. Another factor, which may contribute to the apparent differences observed in the single copy gene complement of some genomes, is that the human genome is often used as a template to identify putative orthologs in metazoan genome projects. This may not be appropriate for some species because of differential evolutionary rates or adaptation, and may lead to orthologs not being identified in the new genomes.

### Assessing genomes and EST datasets

To demonstrate the utility of a complete ortholog dataset for a particular phylogenetic group, we assessed the number of single copy orthologs in 18 established and 6 draft or recently published metazoan genomes (and 1 outgroup genome) (Supplemental Table S4), as well as in 62 published EST datasets (Supplemental Table S5). The percentage of detected single copy orthologs can be used as a good approximation of genome completeness.

**Figure 4:**
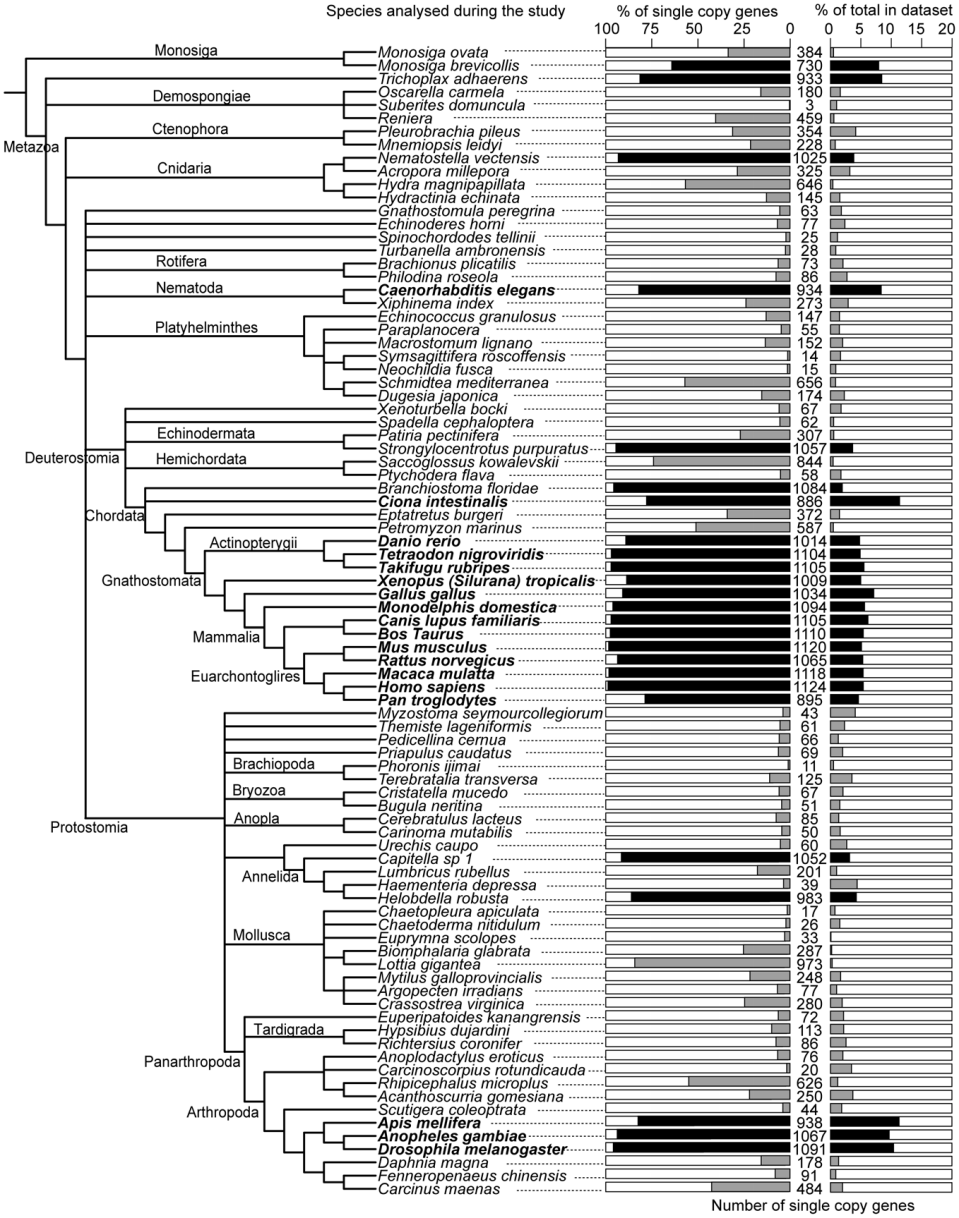
Distribution of single copy genes in the analyzed species. Distribution of single copy genes across all studied species. The tree contains the species analyzed in this study and their relationships as defined by the NCBI taxonomy. The number of single copy genes found in each species is shown, along with a representation of that value as a percentage of all the 1,126 single copy genes and as a percentage of the total number of genes in the genome or EST dataset used. The black bars represent counts from genomes, grey bars from published EST datasets. Species names in bold indicate the species that were used to define the set of single copy orthologs.

The choice of genomes to include is an inherent problem for identifying true single copy orthologs, as one would expect the number of gene losses observed in individual gene families to increase with the number of genomes included in the analysis. In our dataset of 18 established genomes, we observed on average 8% of the genes missing per genome, ranging from only 2 missing genes in human to 370 in *Ciona intestinalis* (Figure 4 and Supplemental Table S1). The large number of missing genes in *Ciona intestinalis* might be due to the divergence of the organism [40], perhaps in combination with incomplete sequencing and/or annotation. As demonstrated above for *Pan trogylodytes*, we expect the number of missing genes to decrease considerably as the quality of the genome annotation increases. For this set of genomes, we would therefore expect on average 1.44 genes/species missing per family, but for a larger dataset of 30 genomes, an average of 2.4 genes missing per family may be more appropriate.

This is further supported by the fact that, on average, only 10% of the single copy orthologs in the 6 draft or recently published metazoan genomes were found to be missing (Supplemental Table S4). While the slightly higher average number of missing single copy orthologs suggests that high quality genomes should be used for the initial definition of orthologous groups, this result demonstrates the universality of these single copy orthologs in the Metazoa.

The majority of the EST datasets examined were far from complete, missing on average 936 (83%) of the 1,126 universal single copy orthologs (ranging from 1,123 (99.8%) missing in *Suberites domuncula* to 153 (14%) in *Lottia gigantea*) (Figure 4 and Supplemental Table S5), even though the datasets contained many thousands of EST sequences (Supplemental Table S5). For instance, there were 164,325 ESTs for *Hydra magnipapillata*, but we failed to identify representatives for 480 (43%) of the single copy orthologs. Despite the different library normalization protocols used for EST dataset generation, the number of single copy orthologs initially correlates with the size of the dataset and then plateaus (Supplemental Figure S4), suggesting that with more data it may be possible to define a minimum number of ESTs necessary to achieve complete coverage of the genes from an organism.

Our results identify taxonomic groups that are poorly represented so far, despite EST sets being available for some species and regardless of coverage and other annotation issues (Figure 4). Among the major groups of Metazoa, the Chordates achieve the best coverage of single copy orthologs, with an average of 989 (88%) per species (the majority of which were genomes). Similarly, on average 1,032 (92%) of the single copy orthologs were found in the Insects (all genomes). However, some other groups were not as well represented: the Crustacea for example had on average 251 (22%) single copy orthologs per dataset (all ESTs), while the Mollusca had 237 (21%). Interestingly, these datasets have been used recently to reconstruct hypotheses about their interrelationships [26], [41]. These major metazoan groups require either representative genomes to be sequenced or in the short-term, larger (or at least normalized) EST datasets to be generated.

### Assessing the method using other resources

Depending on the methods and sequence databases used to construct the orthologous groups, the exact content of a specific gene family can differ [42]. In general, corresponding gene families in different databases will share a “core” of proteins, but the inclusion of differing “peripheral” proteins causes differences to be observed in copy numbers in some species. In order to demonstrate that our results were not just a product of the approach taken to reconstruct the gene families in eggNOG, we also carried out the single-copy ortholog identification using the Ensembl compara dataset (version 56) [16].

The Ensembl compara dataset consists of 18,762 gene families, constructed using 830,582 genes from 49 metazoan species and one fungal species (*Saccharomyces cerevisiae*). Gene sequences and computed phylogenies are provided for each family [16], however as there is no indication of levels of support for each of the internal branches on the computed trees, we reconstructed multiple sequence alignments and phylogenies for each gene family, retaining only the most highly supported branches. This dataset was much larger than the dataset from eggNOG, but included genomes of varying quality and sequencing coverage. Given the influence of these factors [43], [44], we carried out three analyses: firstly with the entire dataset, then excluding all those genomes that had less than 3× coverage and finally less than 7× coverage. The resulting datasets comprised 49, 34 and 24 genomes respectively (Supplemental Figure S5).

Using all 49 genomes, only 112 single-copy gene families were identified using the standard taxon-count approach, while the phylogenetic analysis rescued 5 additional ones, representing an increase of 4.5%. However, when the lowest quality genomes were excluded (less than 3× coverage) the total increased to 258, of which 34 were identified only using our method (a total of 15% increase in single-copy families). Finally, when only the highest quality genomes were included (greater than 7× coverage), we were able to identify a total of 687 single-copy orthologs, of which 173 were found with the phylogenetic approach, increasing the number of single-copy gene families by 34%. This latter number is comparable to the 36% increase achieved with the eggNOG dataset using the same genome quality.

The increased number of identified single copy gene families demonstrates the advantage of analysing multigene families using a phylogenetic approach. Such differentiation between orthology and ‘hidden paralogy’ can only be achieved by taking the phylogenetic signal of the gene family into account, such as in our gene tree reconciliation analysis. This is especially important when marker genes are used for the purposes of reconstructing phylogenetic trees.

### Conclusions

In conclusion, we report a protocol for the identification of single copy orthologs in Metazoa that leads to considerably higher accuracy than other methods. The absence of these genes in some genomes seems to indicate mostly missing sequence and gene annotation rather than true gene loss. This provides a unified, fast and efficient way to estimate the expected number of missing genes in a genomic or transcriptomic dataset. Furthermore, the low numbers of universal single copy metazoan orthologs in current EST datasets point to their relatively low gene coverage, despite the fact that these datasets sometimes contain many thousands of ESTs. The set of metazoan single copy orthologs derived here should not only be useful for simple coverage control of genomic datasets, but with its 600,000 aligned amino acid positions, it represents a dataset which is likely to be very useful for many other phylogenetic studies.

## Materials and Methods

We define the metazoan single copy orthologs as the set of genes that have remained in single copy (without duplications or losses occurring) since the last metazoan common ancestor. All Metazoa should possess these genes and any absence would represent incomplete sampling from the species or misannotation. Marker genes such as these are identified by compiling all the genes that are in “one to one” relationships with orthologs in other species. However, this fails to identify subsets of large-multigene families, which may have remained in single copy since the last common ancestor of the species in question. In order to address this inadequacy, our methodology as outlined in Figure 1 consists of 4 main steps:

1. Construction of a robust eukaryotic species tree
2. Identification of single copy orthologs from the meNOGs
3. Extraction of single copy orthologs from draft genomes and EST datasets
4. Assessing the method using other resources

### 1) Eukaryotic species tree construction (Figure 1.1)

The 40 universal gene families previously described in [35] were used to construct a species tree of the Eukaryotes. Each of the gene families was aligned separately using Muscle [45] with the default settings. Gblocks [46] was then used to remove the badly aligned regions (using the default settings, except for the following: Minimum Length Of A Block = 2; Allowed Gap Positions = all). All 40 resulting Multiple Sequence Alignments (MSA) were manually checked and then concatenated. Next, 100 bootstrap replicates of the alignment were carried out using the SEQBOOT program from the Phylip package [47]. Following this, PhyML [48] was used to find the maximum likelihood tree for each of the 100 bootstrap replicates and for the original alignment. The parameters used were as follows: the JTT model of evolution with the proportion of invariable sites estimated; site rate-heterogeneity was estimated using a gamma model with an estimated alpha parameter; rate heterogeneity was summarized using 4 site categories.

Finally, a consensus tree was constructed, using the CONSENSE program from the Phylip package [47]. The phylogenetic hypotheses constructed were visualized using the iTOL web server [49]. A pruned version of this tree containing only the species in our set of metazoan orthologous groups (e.g. 19 species in the meNOGs including *Monosiga brevicollis* as an outgroup) was extracted from the resulting phylogeny. The resulting pruned tree supported the Coelomata hypothesis of animal evolution. A second version of the tree was constructed by hand which supported the competing Ecdysozoa hypothesis (Figure 3). Both species trees were then used in the subsequent analyses so as not to bias results towards either of the two hypotheses.

### 2) Identification of single copy orthologs from the meNOGs (Figure 1.2)

The metazoan Non-supervised Orthologous Groups (meNOGs) were obtained from the eggNOG database (Version 1) [14]. The meNOGs are gene families built from 363,805 proteins from the following 18 metazoan species: *Homo sapiens, Pan troglodytes, Macaca mulatta, Mus musculus, Rattus norvegicus, Canis familiaris, Bos taurus, Monodelphis domestica, Gallus gallus, Xenopus tropicalis, Tetraodon nigroviridis, Takifugu rubripes, Danio rerio, Ciona intestinalis, Anopheles gambiae, Drosophila melanogaster, Apis mellifera, Caenorhabditis elegans* from version 38 of Ensembl (see: http://www.ensembl.org/info/website/archives/assembly.html for details of the genome versions). The meNOGs link 241,305 proteins in 23,033 gene families. They can be divided into 4,404 groups having a 1-to-many relationship (i.e. only a single species had duplication events), 3,721 many-to-many (i.e. multiple species have undergone duplications) and 14,908 with 1-to-1 relationships (i.e. a single copy for each genome). The single copy relationships between the different numbers of species in eggNOG are outlined in Supplemental Table S6.

#### Identification of single copy orthologs

Using the gene families from eggNOG, we searched for potential metazoan marker genes that have been in single copy since the last common metazoan ancestor. In order to overcome possible misannotation in the genomes used in the analysis, we allowed for the absence of a gene copy if the reconciliation showed it was species-specific. Similarly, we also allowed for the inclusion of a family with a duplication if the duplication event was species-specific (e.g. in some genomes, pseudogenes are not annotated and appear as artificial duplications). For single copy orthologs found in all Metazoa, naturally occurring duplications are rare [50]. The protocol resulted in the identification of 822 single copy genes (219 found in all 18 species, 125 with 1 duplication, 478 with 1 loss) (see Supplemental Table S2 for more details).

We also identified sub-families of large multigene families that had been in single copy since the last common ancestor of the Metazoa. Since we used a phylogenetic approach, we were able to locate duplications or losses in the sub-families. Thus, we only included those sub-trees that had undergone only species-specific duplications or losses, or none at all. The rationale behind the inclusion of these duplications or losses was that they would have no effect on the phylogenetic signal of the metazoan species tree (if indeed they were real duplications or losses and not just misannotations).

Firstly, robust MSA were constructed for each of the 23,033 meNOGs. Of these, 20,262 contained more than 2 sequences and were aligned using the AQUA program [51], which was setup to run Muscle [45] and Rascal [52]. AQUA exploits the NORMD program [51], in order to assess the quality of each individual MSA and to select the best MSA with the highest norMD score. Here, the Muscle MSA was selected in 14,617 of the cases and the refined Rascal MSA in 5,645 of the cases. The distribution of the norMD scores in the resulting 20,262 MSA is a good indicator of the quality of our dataset. Indeed, one can observe in Supplemental Figure S6 the high proportion of highly reliable MSA (i.e. norMD score>0.6 [52]).

Secondly, each of the meNOG alignments was used to construct a phylogenetic tree. This was done by initially carrying out 100 bootstrap replicates of each alignment using the SEQBOOT program from the Phylip package [47]. Following this, PhyML [48] was used to find the maximum likelihood tree for each of the 100 bootstrap replicates and for the original alignment. The parameters used were as follows: the JTT model of evolution with the proportion of invariable sites estimated; site rate-heterogeneity was estimated using a gamma model with an estimated alpha parameter; rate heterogeneity was summarized using 4 site categories. A consensus tree was constructed using the “consensus” command in Clann [53]. In general, sequence format conversion was carried out using the ReadSeq program [54]. The phylogenetic hypotheses constructed were visualized using the iTOL web server [49].

**Figure 5:**
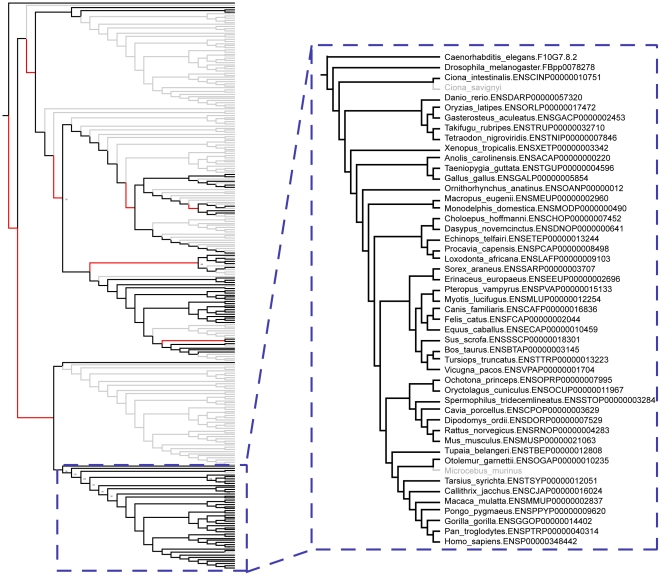
Multigene family reconstruction. An example of the reconciliation of a proteasome 26S subunit multigene family is shown in the left. Duplications are hypothesized to have occurred on the branches colored in red, while those branches that are hypothesized to be lost are in grey. The subtree in the dashed box has been identified as being in single copy. The tree on the right is a more detailed view of the same clade. The leaves on the tree are labeled with their species names followed by the protein ID of the specific sequence that was mapped to that position.

Finally, each of the meNOG trees was reconciled with the two eukaryotic species trees (i.e. Coelomata and Ecdysozoa trees) (Figure 3) using gene-tree parsimony [34] as implemented in Clann (version 4) [53]. This procedure assumes that all conflicts between the gene trees and the species trees arise from either duplications or losses (which is reasonable when dealing with the Metazoa) and estimates the most parsimonious solution for the number of duplications and losses required to explain the discrepancies between them [34], [36]. As gene trees are (by their nature) unrooted and our protocol requires a reliable rooting, this procedure was carried out for every possible rooting of each of the gene trees. The number of duplications and losses calculated for each rooting was used as an indication of the reliability of the rooting. The most parsimonious rooting (which required the fewest number of duplications and losses to explain the difference between its topology and that of the two species trees) was used to study the duplications and losses in the Metazoa (Figure 2). Unresolved internal branches in the gene trees are treated as soft polytomies during the reconciliation process and are assumed not to conflict with the species tree (thus do not contribute to the number of duplications and losses reconstructed). The sub-trees of meNOGs that were in single copy since the last metazoan ancestor were then identified, extracted and classified as single copy orthologs (allowing for species-specific duplication or losses to account for genome annotation errors) (see Figure 5 for an example).

This gene tree reconciliation method identified a further 304 single copy orthologs. Since our approach is dependent upon constructing reliable trees for each of the gene families, (as described above) we summarized the bootstrapped trees, retaining only those relationships with greater than or equal to 80% BP support. Even using this conservative approach, it is possible that phylogenetic reconstruction artifacts, such as systematic bias, long branch attraction or poor model selection, may cause the gene trees to differ from the “species” tree. However, our method excludes any sub-trees that explicitly differ from the species trees (e.g. not having monophyletic chordates). This reduced the number of single copy orthologs identified, but also minimized the possibility of including false positives.

After removal of redundant single copy orthologs found using both of the species trees described above, a total of 1126 meNOGs were used to define our final dataset (see Supplemental Table S3 for details of the genes in the dataset).

In order to explore some of the characteristics of the single copy meNOGs identified here, we report their function distribution (see Supplemental Figure S7), expression profiles (see Supplemental Figure S2) and sequence conservation (Supplemental Figure S3).

#### Functional distribution

The functional classifications for each of the 1,126 single copy genes were extracted from the automatically generated annotations in the eggNOG database [14]. These functions were summarized in 4 categories: Poorly characterized; Metabolism; Cellular processes and signaling; Information storage and processing (Supplemental Figure S7).

#### Gene expression comparison

Gene expression data for 33,675 human gene transcripts from 79 tissue types were downloaded from the BIOGPS database [55]. A subset of 61 transcripts, which overlapped with our dataset of single copy orthologs, was identified from this larger dataset. The average GC-RMA values across all 79 tissue types was calculated and the expression level distribution for this subset of 61 genes was compared to the distribution for all 33,675 genes in our dataset using the R statistical software package [56] (Supplemental Figure S2).

#### Sequence conservation within the meNOGs

To provide an estimate of evolutionary divergence, we calculated the mean percent identity in the MSA for each meNOG (described as the “FamID” in [57]). We then compared the sequence conservation distribution for the 1,126 single copy orthologs to the distribution for the full set of 20,262 meNOGs. No specific differences could be observed, indicating that our dataset of single copy meNOGs contains the full spectrum from fast to slow evolving gene families (Supplemental Figure S3).

### 3) Extraction of single copy orthologs from draft genomes and EST datasets (Figure 1.3)

#### Draft genomes

Six draft metazoan genomes (*Capitella, Trichoplax adhaerens, Branchiostoma floridae, Helobdella robusta, Nematostella vectensis and Strongylocentrotus purpuratus*) and 1 draft outgroup genome (*Monosiga brevicollis*) were assessed for completeness using the 1,126 single copy orthologs (see Supplemental Table S4). All the proteins from these draft genomes were aligned using the PARALIGN software [58] and the Smith-Waterman algorithm against the 363,805 proteins in eggNOG. Genome proteins were assigned to the meNOGs based on best reciprocal hits (with a bit score threshold of at least 180). The number of proteins assigned from each genome to the meNOGs is outlined in Supplemental Table S4. The genes assigned to any of the 1,126 gene families found to be in single copy in the Metazoa were retained.

#### EST datasets

The 62 metazoan EST datasets, described in Supplemental Table S5, were assembled to assess their completeness using the single copy orthologs identified as part of this study. The following procedure was used to extract the single copy orthologs from each of the EST datasets separately.

1. Each EST was aligned to all proteins from eggNOG, using the BLASTX program from the Washington University's BLAST package (WUBLAST) (http://blast.wustl.edu). Alignments with a bit score greater than or equal to 60 bits were considered significant and were retained for further analysis.
2. Each EST with significant alignments to proteins belonging to single copy orthologous groups were extracted. Any ESTs with a higher affinity to a protein that was not a member of the single copy gene families was discarded in order to minimize the possibility of including paralogs. Generally, for each EST related to a single copy orthologous group, multiple significant alignments to different family members were found. This information was used to identify the first and last position on the EST that matched the orthologous group. These positions were then extended where possible to the nearest methionine or stop codon respectively. Finally, this portion of the EST was extracted and translated into its amino acid equivalent using the reading frame indicated from the BLASTX results. These ESTs are referred to hereafter as the 1-to-1 ESTs.
3. In order to identify ESTs that may be mitochondrial versions of genes included in the single copy orthologous groups, a database of 1,016 mitochondrial genomes was retrieved from NCBI RefSeq [59]. Alignments were then carried out between the 1-to-1 ESTs and all the mitochondrial sequences, using the BLASTP program from the WUBLAST package. ESTs that aligned to a mitochondrial sequence with a bit score equal or higher than the best bit score from the genomic databases were discarded.
4. All remaining single copy ESTs were combined with the sequences from the meNOGs to which they belonged. MSAs for each family were then computed using the default settings in Muscle [45].
5. Using the aligned sequences, multiple ESTs were then assembled into a single sequence by combining ESTs that spanned different parts of the gene and discarding ESTs that represented portions of the gene covered by larger ESTs.
6. Finally, the quality of the assembled ESTs was assessed at the level of the whole sequence and at the level of individual assembled sites, using the following two methods:
  - The quality of the combined and translated EST sequences was assessed by aligning them individually to each of the sequences from its single copy ortholog family, using the BLASTP program from the WUBLAST package. ESTs with similarity scores of less than 60 bits to the best-matching single copy ortholog were discarded. This filter was designed to remove ESTs that were not translated into the correct amino acid equivalent, generally due to sequencing errors changing the reading frame mid-sequence when several ESTs were assembled into a single sequence.
  - Individual sites of the assembled and translated EST sequences were assessed using a Hidden Markov Model (HMM) based on the genomic sequences corresponding to its meNOG, using HMMBUILD from the HMMer package [60]. Each assembled EST was then aligned to the HMM using HMMALIGN [60] and sites in the combined ESTs that did not align to the HMM were discarded. This filter was designed to remove sites that were originally at the start or end of an individual EST (but did not belong to the coding sequence), and that were relocated within the combined sequence during the EST assembly process (step 5 above).

### 4) Assessing the method using other resources

Metazoan gene families and their associated sequences were retrieved from the ENSEMBL compara (Version 59) database [16]. The ENSEMBL compara dataset consists of 18,762 gene families, constructed using 830,582 genes from 49 metazoan species and one fungal species (*Saccharomyces cerevisiae*) as an outgroup. While phylogenies for each of the gene families are provided, there is no indication of the support level for each internal branch. In order to include only the most highly supported hypotheses of relationships, we extracted the sequences for all the proteins in a given gene family and realigned them using AQUA [51]. The resulting alignment was then used to build a phylogeny from 100 bootstrap resamplings using BIONJ [61] in Paup* [62]. The representative species tree provided by ENSEMBL for these genomes was used for the purposes of the reconciliation analysis.

To study the effect of including genomes of varying quality, we identified the levels of coverage of the ENSEMBL genomes. We then carried out three analyses: the first included all the metazoan genomes from the dataset; the second excluded those genomes with less than 3× coverage; the third excluded those genomes with less than 7× coverage. This resulted in datasets containing 49, 34 and 24 genomes respectively. For each dataset, all 18,762 gene trees, as well as the Ensembl species tree, were pruned down to the corresponding taxon set. A standard taxon-count approach was then used to identify the number of single copy gene families in each dataset. For the remaining multigene families, Clann [53] was used to perform gene tree reconciliations in order to identify sub-trees that were in single copy in the Metazoa.

## Supporting Information

Figure S1 **Distribution of average gene lengths.** The distribution of average gene lengths (in amino acids) of the 1,126 single copy metazoan orthologs identified as part of this analysis.
(PDF)

Figure S2 **Comparison of the expression profiles of the single copy orthologs with all known human transcripts.** The average GC-RMA normalized expression profiles of 33,675 human gene transcripts from across 79 tissue types are compared with the expression profiles of the 61 single copy orthologs for which we could find expression profiles from the same tissue types. The expression profile data was retrieved from the BioGPS database [55].
(PDF)

Figure S3 **Comparison of the mean percent identities of the single copy orthologs with all orthologous groups.** The distributions of the mean percent identities for the 20,262 orthologous groups in the meNOGs and for the 1,126 single copy orthologs identified as part of this study.
(PDF)

Figure S4 **Statistics of the EST datasets analyzed in this study.** A) The number of core metazoan gene families found versus the number of ESTs in the dataset. B) The average size of a gene versus the number of EST datasets in which it was found.
(PDF)

Figure S5 **ENSEMBL compara (version 59) genomes.** The genomes from ENSEMBL version 59 used to demonstrate the effectiveness of the reconciliation technique on another dataset. The boxes indicate the level of coverage that the genome sequence had reached at this version.
(PDF)

Figure S6 **Distribution of norMD scores calculated.** Distribution of norMD scores computed for the MSAs of the 20,262 meNOGs.
(PDF)

Figure S7 **Functional classifications of the genes in the single copy gene dataset.** The bar chart shows the functional classifications for all 1,126 single copy gene families.
(PDF)

Table S1 **Genomes used to define the orthologous groups.** The genomes used to define the orthologous groups, from which single copy orthologs in Metazoa were identified.
(PDF)

Table S2 **Number of single copy gene families identified using the taxon-count approach.** The number of gene families identified as having either a single loss or duplication in an individual metazoan species, using the standard taxon-count approach.
(PDF)

Table S3 **Description of all 1,126 single copy orthologs identified.** Gene descriptions where available for the 1,126 single copy orthologous groups identified as part of this study.
(PDF)

Table S4 **Details of the draft or recently published genomes assessed.** NCBI = National Center for Biotechnology Information. JGI = Joint Genome Institute (These sequence data were produced by the US Department of Energy Joint Genome Institute http://www.jgi.doe.gov/ in collaboration with the user community).
(PDF)

Table S5 **EST datasets assessed for completeness.** The EST datasets assessed for completeness as part of this study.
(PDF)

Table S6 **Distribution of single copy meNOGs according to species composition.** The distribution of single copy meNOGs according to species composition.
(PDF)
