# Supplementary figures and images for: Identifying Single Copy Orthologs in Metazoa
Source: PLoS Comput Biol. 2011 Dec 1;7(12):e1002269. doi: 10.1371/journal.pcbi.1002269 (PMC3228760; doi:10.1371/journal.pcbi.1002269)

# Distribution of lengths of single-copy metazoan orthologs

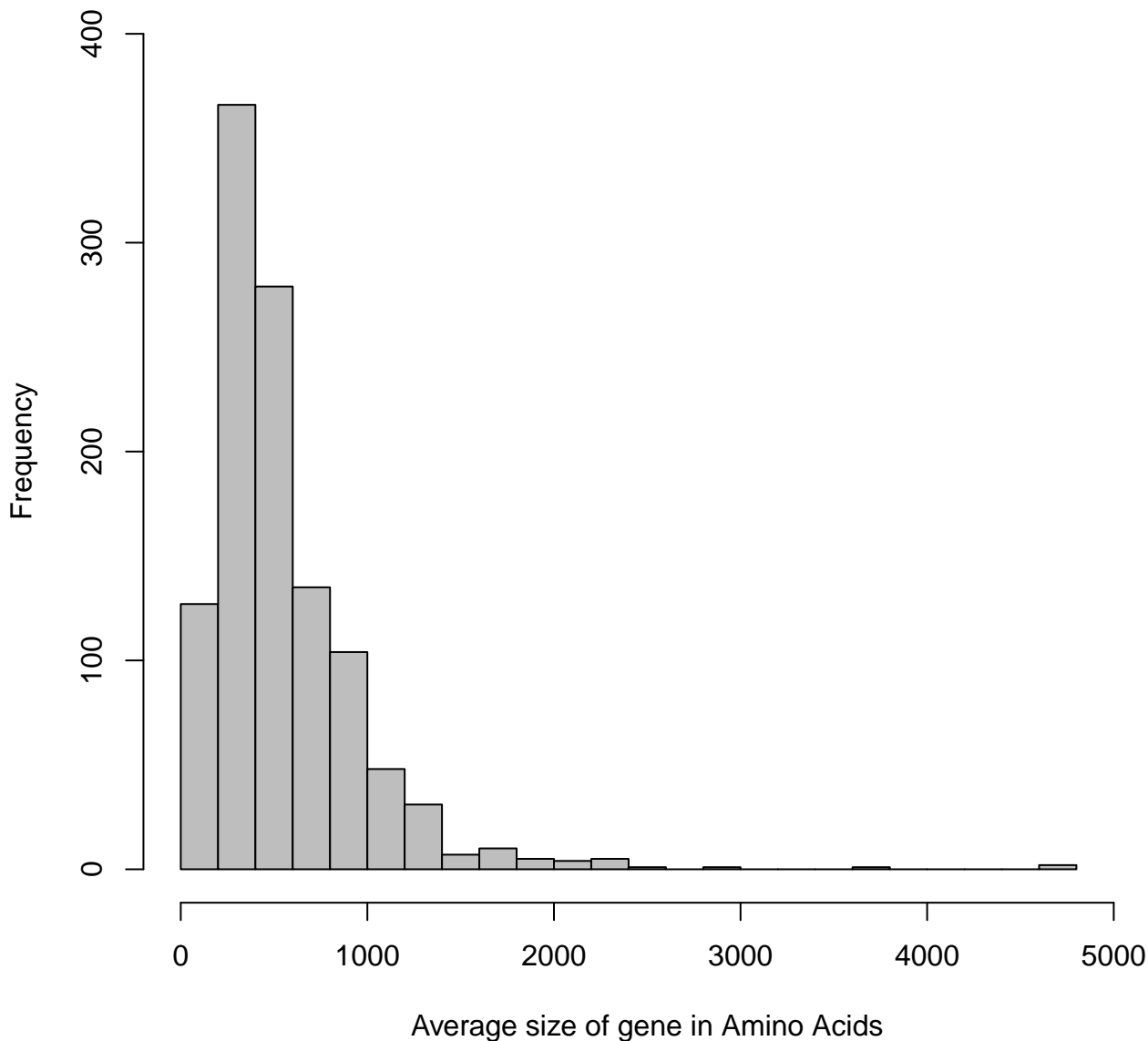

Supplement: Figure S1 — Distribution of average gene lengths. The distribution of average gene lengths (in amino acids) of the 1,126 single copy metazoan orthologs identified as part of this analysis. (PDF) [file pcbi.1002269.s001.pdf]

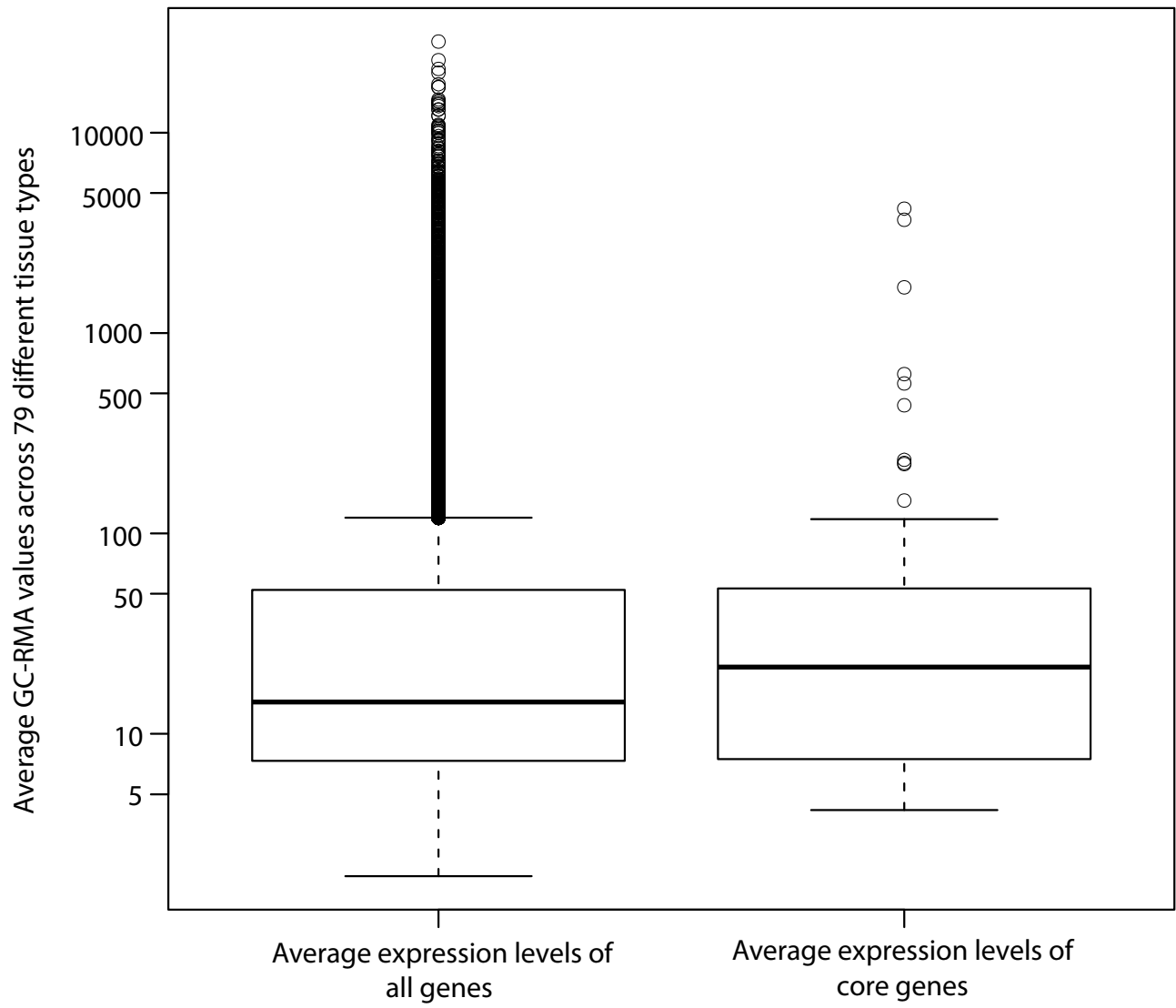

Supplement: Figure S2 — Comparison of the expression profiles of the single copy orthologs with all known human transcripts. The average GC-RMA normalized expression profiles of 33,675 human gene transcripts from across 79 tissue types are compared with the expression profiles of the 61 single copy orthologs for which we could find expression profiles from the same tissue types. The expression profile data was retrieved from the BioGPS database [55]. (PDF) [file pcbi.1002269.s002.pdf]

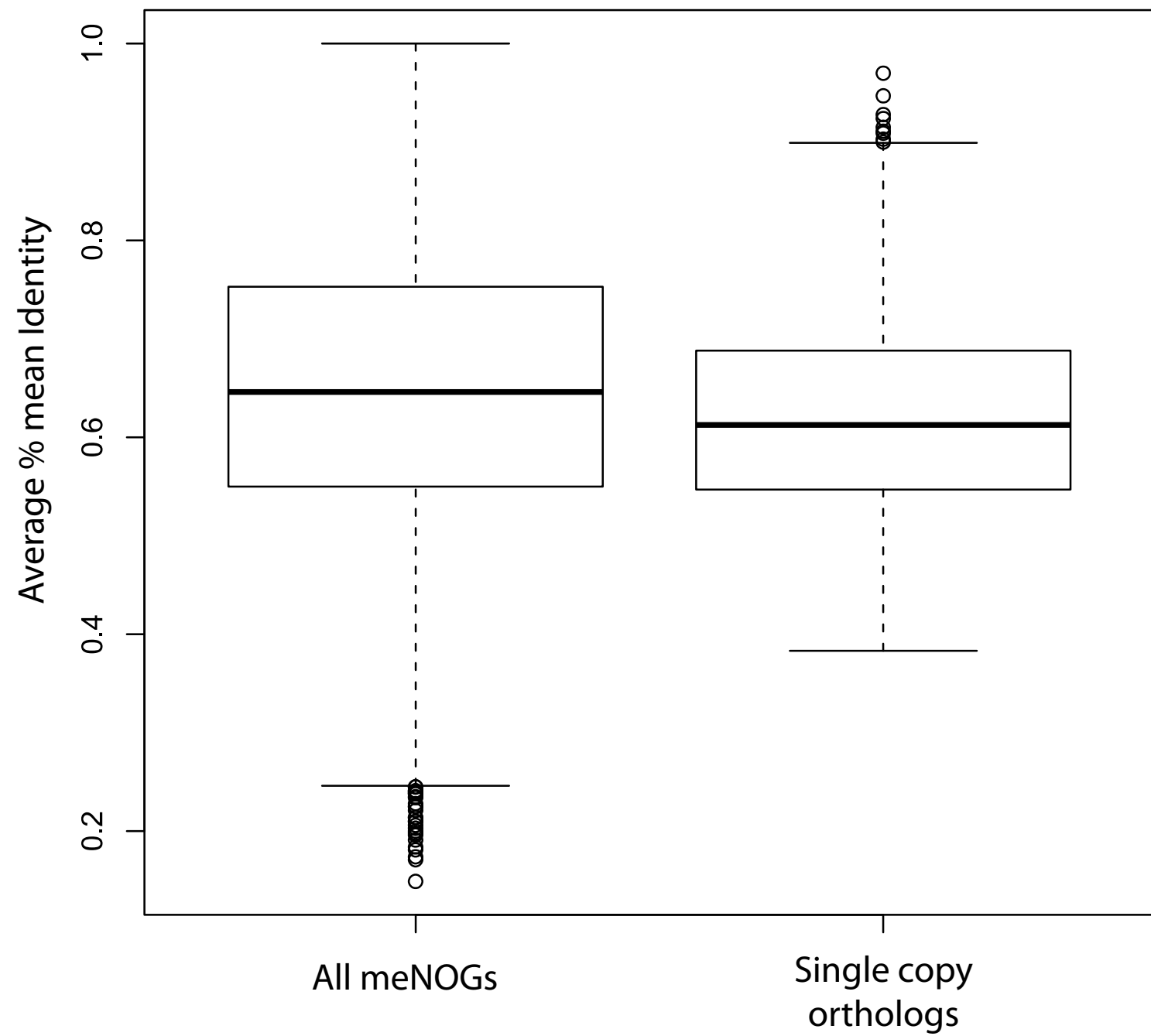

Supplement: Figure S3 — Comparison of the mean percent identities of the single copy orthologs with all orthologous groups. The distributions of the mean percent identities for the 20,262 orthologous groups in the meNOGs and for the 1,126 single copy orthologs identified as part of this study. (PDF) [file pcbi.1002269.s003.pdf]

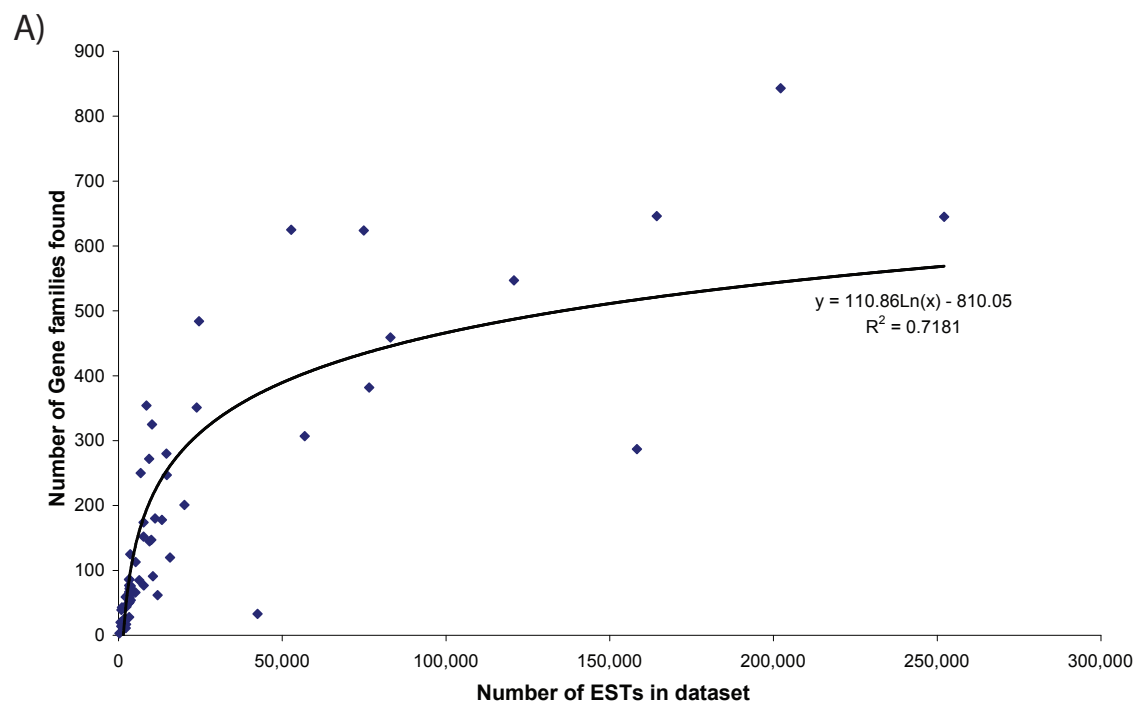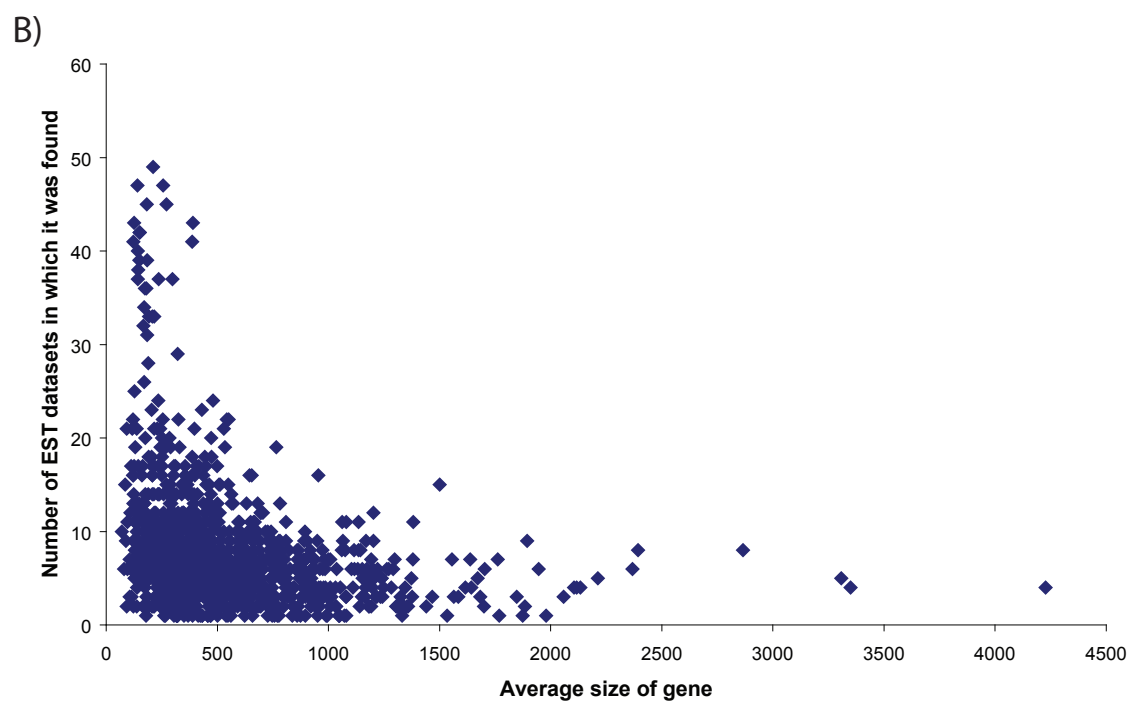

Supplement: Figure S4 — Statistics of the EST datasets analyzed in this study. A) The number of core metazoan gene families found versus the number of ESTs in the dataset. B) The average size of a gene versus the number of EST datasets in which it was found. (PDF) [file pcbi.1002269.s004.pdf]

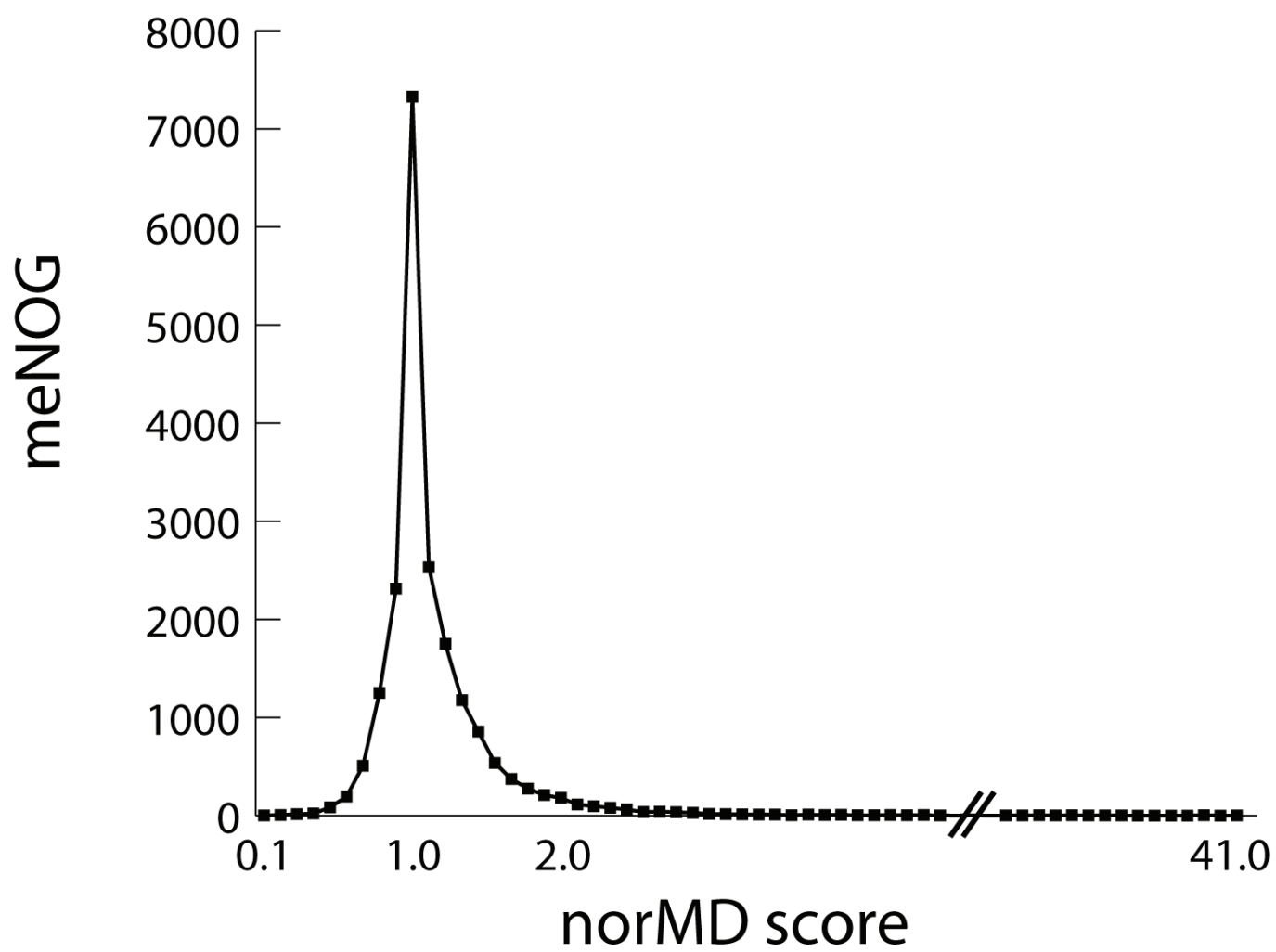

Supplement: Figure S6 — Distribution of norMD scores calculated. Distribution of norMD scores computed for the MSAs of the 20,262 meNOGs. (PDF) [file pcbi.1002269.s006.pdf]
