# Supplementary material for: Identifying Single Copy Orthologs in Metazoa
Source: PLoS Comput Biol. 2011 Dec 1;7(12):e1002269. doi: 10.1371/journal.pcbi.1002269 (PMC3228760; doi:10.1371/journal.pcbi.1002269)
Supplement: Figure S5 — ENSEMBL compara (version 59) genomes. The genomes from ENSEMBL version 59 used to demonstrate the effectiveness of the reconciliation technique on another dataset. The boxes indicate the level of coverage that the genome sequence had reached at this version. (PDF) [file pcbi.1002269.s005.pdf]

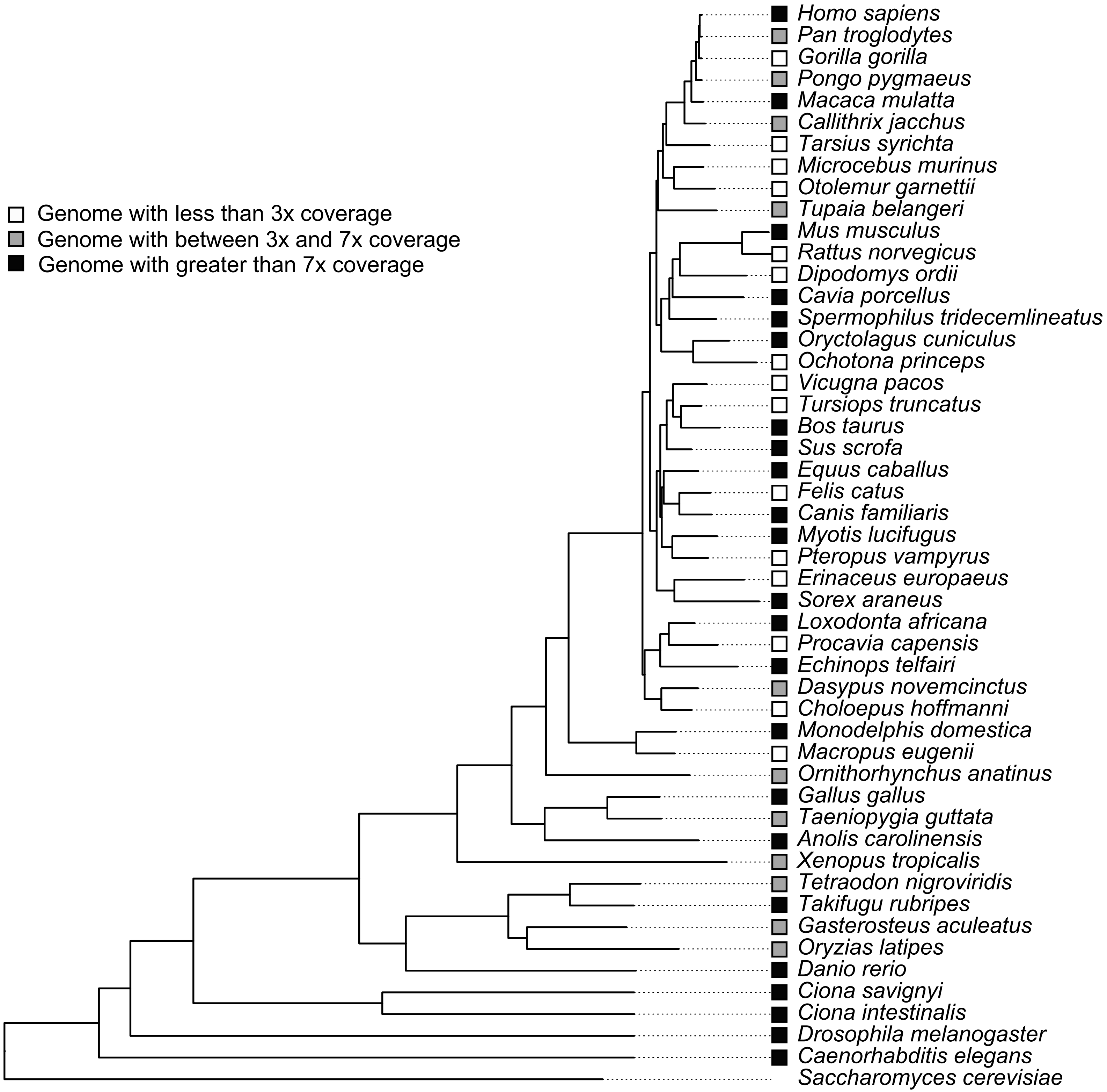

Genome with less than 3x coverage
Genome with between 3x and 7x coverage
Genome with greater than 7x coverage
Homo sapiens
Pan troglodytes
Gorilla gorilla
Pongo pygmaeus
Macaca mulatta
Callithrix jacchus
Tarsius syrichta
Microcebus murinus
Otolemur garnettii
Tupaia belangeri
Mus musculus
Rattus norvegicus
Dipodomys ordii
Cavia porcellus
Spermophilus tridecemlineatus
Oryctolagus cuniculus
Ochotona princeps
Vicugna pacos
Tursiops truncatus
Bos taurus
Sus scrofa
Equus caballus
Felis catus
Canis familiaris
Myotis lucifugus
Pteropus vampyrus
Erinaceus europaeus
Sorex araneus
Loxodonta africana
Procavia capensis
Echinops telfairi
Dasypus novemcinctus
Choloepus hoffmanni
Monodelphis domestica
Macropus eugenii
Ornithorhynchus anatinus
Gallus gallus
Taeniopygia guttata
Anolis carolinensis
Xenopus tropicalis
Tetraodon nigroviridis
Takifugu rubripes
Gasterosteus aculeatus
Oryzias latipes
Danio rerio
Ciona savignyi
Ciona intestinalis
Drosophila melanogaster
Caenorhabditis elegans
Saccharomyces cerevisiae
