# Supplementary material for: Identifying Single Copy Orthologs in Metazoa
Source: PLoS Comput Biol. 2011 Dec 1;7(12):e1002269. doi: 10.1371/journal.pcbi.1002269 (PMC3228760; doi:10.1371/journal.pcbi.1002269)
Supplement: Figure S7 — Functional classifications of the genes in the single copy gene dataset. The bar chart shows the functional classifications for all 1,126 single copy gene families. (PDF) [file pcbi.1002269.s007.pdf]

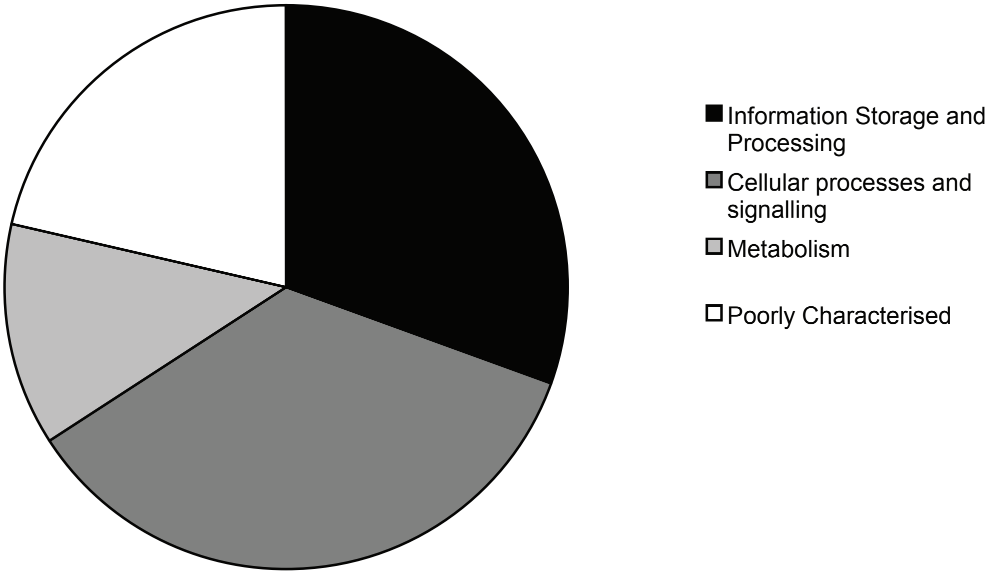
Information Storage and Processing
Cellular processes and signalling
Metabolism
Poorly Characterised
