# Supplementary material for: Identifying Single Copy Orthologs in Metazoa
Source: PLoS Comput Biol. 2011 Dec 1;7(12):e1002269. doi: 10.1371/journal.pcbi.1002269 (PMC3228760; doi:10.1371/journal.pcbi.1002269)
Supplement: Table S1 — Genomes used to define the orthologous groups. The genomes used to define the orthologous groups, from which single copy orthologs in Metazoa were identified. (PDF) [file pcbi.1002269.s008.pdf]

TaxID	Name	Genome publication date	Number of Genes	Number of Single copy orthologs	% of total single copy orthologs
6239	*Caenorhabditis elegans*	11/12/1998	11,187	934	83
7165	*Anopheles gambiae*	04/10/2002	11,038	1,067	95
7227	*Drosophila melanogaster*	24/03/2000	10,514	1,091	97
7460	*Apis mellifera*	01/11/2006	8,300	938	83
7719	*Ciona intestinalis*	13/12/2002	7,791	886	79
7955	*Danio rerio*	05/07/2005	21,234	1,014	90
8364	*Xenopus tropicalis*	01/08/2005	20,270	1,009	90
9031	*Gallus gallus*	09/12/2004	14,533	1,034	92
9544	*Macaca mulatta*	13/04/2007	20,791	1,118	99
9598	*Pan troglodytes*	01/09/2005	19,474	895	79
9606	*Homo sapiens*	15/02/2001	20,809	1,124	99
9615	*Canis familiaris*	08/12/2005	17,919	1,105	98
9913	*Bos taurus*	01/06/2005	20,565	1,110	99
10090	*Mus musculus*	05/12/2002	22,011	1,120	99
10116	*Rattus norvegicus*	01/04/2004	20,017	1,065	95
13616	*Monodelphis domestica*	10/05/2007	19,490	1,094	97
31033	*Takifugu rubripes*	23/08/2002	20,093	1,105	98
99883	*Tetraodon nigroviridis*	21/10/2004	22,627	1,104	98
