# Supplementary material for: Identifying Single Copy Orthologs in Metazoa
Source: PLoS Comput Biol. 2011 Dec 1;7(12):e1002269. doi: 10.1371/journal.pcbi.1002269 (PMC3228760; doi:10.1371/journal.pcbi.1002269)
Supplement: Table S2 — Number of single copy gene families identified using the taxon-count approach. The number of gene families identified as having either a single loss or duplication in an individual metazoan species, using the standard taxon-count approach. (PDF) [file pcbi.1002269.s009.pdf]

TaxID	Name	Loss	Dup
6239	*Caenorhabditis elegans*	57	4
7165	*Anopheles gambiae*	13	7
7227	*Drosophila melanogaster*	2	9
7460	*Apis mellifera*	59	5
7719	*Ciona intestinalis*	78	4
7955	*Danio rerio*	30	44
8364	*Xenopus tropicalis*	19	3
9031	*Gallus gallus*	25	5
9544	*Macaca mulatta*	4	3
9598	*Pan troglodytes*	126	0
9606	*Homo sapiens*	0	2
9615	*Canis familiaris*	6	5
9913	*Bos taurus*	8	7
10090	*Mus musculus*	0	4
10116	*Rattus norvegicus*	36	1
13616	*Monodelphis domestica*	3	3
31033	*Takifugu rubripes*	7	12
99883	*Tetraodon nigroviridis*	5	7
	**Total**	478	125
