# Supplementary material for: Identifying Single Copy Orthologs in Metazoa
Source: PLoS Comput Biol. 2011 Dec 1;7(12):e1002269. doi: 10.1371/journal.pcbi.1002269 (PMC3228760; doi:10.1371/journal.pcbi.1002269)
Supplement: Table S3 — Description of all 1,126 single copy orthologs identified. Gene descriptions where available for the 1,126 single copy orthologous groups identified as part of this study. (PDF) [file pcbi.1002269.s010.pdf]

meNOG ID	Average length of gene (AA)	eggNOG Human protein ID	Annotated Function
meNOG04004	965	9606.ENSP00000344003	Protein involved in cell-cell adhesion
meNOG04006	407	9606.ENSP00000311430	Ribosomal protein L4
meNOG04008	395	9606.ENSP00000262876	Cysteine desulfurase, mitochondrial precursor (EC 2.8.1.7)
meNOG04013	257	9606.ENSP00000262584	Ribosomal protein
meNOG04014	2197	9606.ENSP00000354587	Fkbp12-Rapamycin complex-associated protein
meNOG04017	416	9606.ENSP00000264221	Phosphoribosylaminoimidazole carboxylase, phosphoribosylaminoimidazole succinocarboxamide
meNOG04019	571	9606.ENSP00000323811	
meNOG04026	310	9606.ENSP00000225665	Mitochondrial 2-oxoglutarate/malate carrier protein (OGCP)
meNOG04029	1132	9606.ENSP00000355168	
meNOG04037	574	9606.ENSP00000263239	Atp-Dependent RNA helicase DDX18 (EC 3.6.1.-)
meNOG04039	1274	9606.ENSP00000324573	Flightless I homolog (Drosophila) protein
meNOG04058	591	9606.ENSP00000339517	
meNOG04059	1220	9606.ENSP00000278065	Protein kinase
meNOG04061	350	9606.ENSP00000307241	
meNOG04067	460	9606.ENSP00000313643	Mitogen-Activated protein kinase
meNOG04069	563	9606.ENSP00000303552	
meNOG04073	1164	9606.ENSP00000311135	Probable ATP-dependent helicase DHX37
meNOG04077	838	9606.ENSP00000263115	Tuftelin interacting protein 11
meNOG04080	542	9606.ENSP00000280326	TCP-1 epsilon protein
meNOG04084	382	9606.ENSP00000271628	Splicing factor 3b, subunit 4
meNOG04086	710	9606.ENSP00000352678	
meNOG04092	1037	9606.ENSP00000273814	Diacylglycerol kinase, theta
meNOG04097	917	9606.ENSP00000349877	Probable cation-transporting ATPase 13A1
meNOG04103	547	9606.ENSP00000235391	60 kDa SS-A/Ro ribonucleoprotein (60 kDa Ro protein)
meNOG04117	947	9606.ENSP00000353576	Nuclear transcription factor, X-box binding 1
meNOG04120	430	9606.ENSP00000262542	
meNOG04126	919	9606.ENSP00000309474	
meNOG04132	723	9606.ENSP00000348587	Nucleolar and coiled-body phosphoprotein 1
meNOG04137	923	9606.ENSP00000271131	
meNOG04138	3743	9606.ENSP00000011700	
meNOG04142	297	9606.ENSP00000273064	
meNOG04143	1107	9606.ENSP00000312735	
meNOG04148	1329	9606.ENSP00000296477	Steroid receptor-interacting SNF2 domain protein
meNOG04149	1093	9606.ENSP00000263141	Eukaryotic translation initiation factor 3 subunit 10 (eIF-3 theta)
meNOG04153	434	9606.ENSP00000318297	Ruvb-Like 1 protein
meNOG04155	405	9606.ENSP00000281525	Tryptophan pyrrolase (Tryptophanase)
meNOG04166	657	9606.ENSP00000337265	Oxysterol-Binding protein-like protein 9
meNOG04168	855	9606.ENSP00000226578	Beta-Mannosidase precursor (EC 3.2.1.25)
meNOG04170	708	9606.ENSP00000342100	
meNOG04172	783	9606.ENSP00000230124	non supervised orthologous group
meNOG04176	2219	9606.ENSP00000261312	Hect domain containing protein 1
meNOG04177	915	9606.ENSP00000261657	non supervised orthologous group
meNOG04185	512	9606.ENSP00000344622	PRP4 pre-mRNA processing factor 4 homolog
meNOG04187	373	9606.ENSP00000245969	
meNOG04189	656	9606.ENSP00000297185	Stress-70 protein, mitochondrial precursor (75 kDa glucose regulated protein)
meNOG04195	1215	9606.ENSP00000277783	
meNOG04200	560	9606.ENSP00000259149	
meNOG04205	1776	9606.ENSP00000297954	Serine/Threonine-Protein kinase WNK1 (EC 2.7.1.37)
meNOG04207	808	9606.ENSP00000353247	
meNOG04214	501	9606.ENSP00000234677	Seryl-Aminoacyl-Trna synthetase
meNOG04219	586	9606.ENSP00000296603	non supervised orthologous group
meNOG04228	628	9606.ENSP00000262545	
meNOG04230	759	9606.ENSP00000278072	non supervised orthologous group
meNOG04233	436	9606.ENSP00000292644	26S protease regulatory subunit 7

meNOG04237	1108	9606.ENSP00000261574	
meNOG04238	601	9606.ENSP00000265245	
meNOG04245	1104	9606.ENSP00000314444	
meNOG04247	549	9606.ENSP00000236959	
meNOG04248	511	9606.ENSP00000263222	
meNOG04250	784	9606.ENSP00000265414	CDC5 cell division cycle 5 protein
meNOG04252	599	9606.ENSP00000216129	
meNOG04255	453	9606.ENSP00000238561	Kinase
meNOG04256	607	9606.ENSP00000300107	Atp-Dependent Clp protease ATP-binding subunit ClpX-like, mitochondrial
meNOG04259	457	9606.ENSP00000297902	
meNOG04269	534	9606.ENSP00000330813	
meNOG04289	708	9606.ENSP00000293739	non supervised orthologous group
meNOG04292	733	9606.ENSP00000286050	
meNOG04295	749	9606.ENSP00000323377	
meNOG04297	728	9606.ENSP00000298875	Cleavage and polyadenylation specificity factor, 100 kDa subunit (CPSF 100 kDa subunit)
meNOG04300	573	9606.ENSP00000262875	
meNOG04301	572	9606.ENSP00000355086	
meNOG04305	1173	9606.ENSP00000313490	Phosphoribosylformylglycinamidine synthase (EC 6.3.5.3)
meNOG04306	819	9606.ENSP00000301364	
meNOG04308	459	9606.ENSP00000348442	
meNOG04311	1404	9606.ENSP00000280379	Protein involved in transport
meNOG04313	698	9606.ENSP00000268171	Furin precursor (EC 3.4.21.75)
meNOG04317	459	9606.ENSP00000354340	Ubiquitin-Activating enzyme E1C
meNOG04322	406	9606.ENSP00000346001	Ribosomal protein L3
meNOG04328	938	9606.ENSP00000308179	
meNOG04331	1121	9606.ENSP00000263331	
meNOG04342	509	9606.ENSP00000216038	
meNOG04344	528	9606.ENSP00000326411	Cwf19-Like 1, cell cycle control (S. pombe) protein
meNOG04348	1607	9606.ENSP00000260818	Dnaj homolog subfamily C member 13 protein
meNOG04350	2269	9606.ENSP00000261637	
meNOG04357	726	9606.ENSP00000262652	Kinesin-Like protein KIF3B
meNOG04359	315	9606.ENSP00000327070	Malate dehydrogenase, mitochondrial precursor
meNOG04361	540	9606.ENSP00000283977	
meNOG04362	475	9606.ENSP00000216774	Signal recognition particle 54 protein
meNOG04364	1272	9606.ENSP00000257342	
meNOG04365	591	9606.ENSP00000281828	
meNOG04371	902	9606.ENSP00000234170	Ccaat/Enhancer-Binding protein zeta
meNOG04373	1170	9606.ENSP00000296266	
meNOG04380	558	9606.ENSP00000350558	
meNOG04381	2214	9606.ENSP00000272845	
meNOG04392	1242	9606.ENSP00000339845	
meNOG04394	643	9606.ENSP00000231572	Arginyl-Trna synthetase
meNOG04395	352	9606.ENSP00000277070	
meNOG04397	344	9606.ENSP00000318351	
meNOG04404	845	9606.ENSP00000290158	
meNOG04411	383	9606.ENSP00000302916	
meNOG04422	2248	9606.ENSP00000005905	
meNOG04429	666	9606.ENSP00000263836	
meNOG04439	671	9606.ENSP00000307491	
meNOG04441	1318	9606.ENSP00000341382	Structural maintenance of chromosomes 4-like 1 protein (Chromosome-associated polypeptide C)
meNOG04452	648	9606.ENSP00000229416	
meNOG04455	576	9606.ENSP00000249270	Zuotin-Related factor 1 (M-phase phosphoprotein 11)
meNOG04462	310	9606.ENSP00000231504	Serine/Threonine protein phosphatase 2A, catalytic subunit
meNOG04481	349	9606.ENSP00000299518	
meNOG04483	1677	9606.ENSP00000261266	Protein-Tyrosine phosphatase 10D
meNOG04486	512	9606.ENSP00000314649	Nad(+)-Dependent succinic semialdehyde dehydrogenase
meNOG04503	568	9606.ENSP00000304370	

meNOG04526	413	9606.ENSP00000347408	Adapter-Related protein complex 3 mu 1 subunit (Mu-adaptin 3A)
meNOG04531	420	9606.ENSP00000248430	Mitochondrial chaperone BCS1 (BCS1-like protein)
meNOG04542	780	9606.ENSP00000216254	Aconitase
meNOG04547	978	9606.ENSP00000216297	Suppressor of Ty 16 homolog (S. cerevisiae) protein
meNOG04553	4751	9606.ENSP00000251742	Protein involved in regulation of protein complex assembly
meNOG04554	825	9606.ENSP00000328690	Atp-Dependent RNA helicase DDX24
meNOG04562	755	9606.ENSP00000295561	
meNOG04566	428	9606.ENSP00000298852	
meNOG04569	2077	9606.ENSP00000320949	non supervised orthologous group
meNOG04574	705	9606.ENSP00000329127	Protein kinase C eta type EC
meNOG04576	927	9606.ENSP00000262027	Trna synthetase
meNOG04585	411	9606.ENSP00000307481	
meNOG04586	417	9606.ENSP00000260985	Isocitrate dehydrogenase [NADP] cytoplasmic
meNOG04587	509	9606.ENSP00000334553	Peptidyl-Prolyl cis-trans isomerase like 2
meNOG04589	734	9606.ENSP00000346781	
meNOG04590	1254	9606.ENSP00000283684	
meNOG04591	797	9606.ENSP00000337518	Protein involved in rRNA metabolism
meNOG04594	816	9606.ENSP00000265849	
meNOG04600	943	9606.ENSP00000264214	non supervised orthologous group
meNOG04602	1101	9606.ENSP00000343535	
meNOG04604	467	9606.ENSP00000247003	
meNOG04606	584	9606.ENSP00000330349	Dead-Box protein abstrakt homolog
meNOG04621	403	9606.ENSP00000261192	Branched-Chain-Amino-Acid aminotransferase, cytosolic (EC 2.6.1.42) BCAT
meNOG04623	803	9606.ENSP00000306761	non supervised orthologous group
meNOG04629	1216	9606.ENSP00000268482	
meNOG04635	738	9606.ENSP00000261776	non supervised orthologous group
meNOG04637	824	9606.ENSP00000288490	
meNOG04640	677	9606.ENSP00000257985	
meNOG04644	517	9606.ENSP00000343111	Nucleolar protein 5A
meNOG04645	552	9606.ENSP00000297151	
meNOG04647	303	9606.ENSP00000263639	
meNOG04662	372	9606.ENSP00000261755	Fumarylacetoacetase
meNOG04663	1043	9606.ENSP00000352418	
meNOG04664	636	9606.ENSP00000265302	NADPH cytochrome P450 reductase
meNOG04677	1935	9606.ENSP00000343745	
meNOG04681	558	9606.ENSP00000264874	
meNOG04683	566	9606.ENSP00000355050	
meNOG04686	1290	9606.ENSP00000280562	Phosphatidylinositol transporter protein
meNOG04688	808	9606.ENSP00000264122	Signal transduction protein CBL-B
meNOG04692	837	9606.ENSP00000228264	DEAD/H (Asp-Glu-Ala-Asp/His) box polypeptide 11
meNOG04695	866	9606.ENSP00000351947	
meNOG04696	916	9606.ENSP00000260363	
meNOG04701	1042	9606.ENSP00000301764	
meNOG04706	840	9606.ENSP00000222418	
meNOG04716	958	9606.ENSP00000325377	Protein involved in phosphate transport
meNOG04719	482	9606.ENSP00000216194	Adenylosuccinate lyase
meNOG04720	1200	9606.ENSP00000251879	
meNOG04723	405	9606.ENSP00000355349	Alpha-N-Acetylgalactosaminidase precursor
meNOG04724	469	9606.ENSP00000262860	Kynurenine 3-monooxygenase (kynurenine 3-hydroxylase)
meNOG04726	657	9606.ENSP00000287862	Carnitine palmitoyltransferase II
meNOG04727	553	9606.ENSP00000216190	
meNOG04728	508	9606.ENSP00000207870	Xylulokinase homolog (H. influenzae)
meNOG04732	789	9606.ENSP00000307387	Programmed cell death 6 interacting protein
meNOG04733	698	9606.ENSP00000216122	
meNOG04736	1147	9606.ENSP00000354047	
meNOG04741	469	9606.ENSP00000325548	CNDP dipeptidase 2
meNOG04744	313	9606.ENSP00000219313	26S proteasome non-ATPase regulatory subunit 7 (26S proteasome regulatory subunit rpn8)

meNOG04746	572	9606.ENSP00000334612	Protein involved in cell proliferation
meNOG04752	684	9606.ENSP00000246957	Receptor-Associated protein
meNOG04754	437	9606.ENSP00000292807	
meNOG04756	1065	9606.ENSP00000274562	Leucyl-Trna synthetase, cytoplasmic
meNOG04772	402	9606.ENSP00000348565	Ubiquitin-Activating enzyme E1-domain containing
meNOG04788	1303	9606.ENSP00000265335	DNA repair protein RAD50 (EC 3.6.-.-)
meNOG04790	556	9606.ENSP00000239585	Tcp-1-Alpha (CCT-alpha) protein
meNOG04793	755	9606.ENSP00000299767	Endoplasmin precursor (Heat shock protein 90 kDa beta member 1)
meNOG04794	297	9606.ENSP00000306043	Cell division control protein 2 homolog (EC 2.7.1.37)
meNOG04796	1141	9606.ENSP00000306830	Ubiquitin carboxyl-terminal hydrolase 47 (EC 3.1.2.15) Ubiquitin
meNOG04800	647	9606.ENSP00000353168	
meNOG04802	1213	9606.ENSP00000310700	Atp-Dependent RNA helicase A (EC 3.6.1.-)
meNOG04806	470	9606.ENSP00000320309	Phenylalanyl-Trna synthetase alpha chain (EC 6.1.1.20)
meNOG04808	868	9606.ENSP00000335193	Vacuolar protein sorting 39 (yeast)
meNOG04809	408	9606.ENSP00000235894	Dolichyl-Diphosphooligosaccharide--Protein glycosyltransferase 48 kDa subunit precursor (EC 2.4.1.119)
meNOG04810	493	9606.ENSP00000231420	
meNOG04812	539	9606.ENSP00000258091	Eta subunit
meNOG04814	469	9606.ENSP00000227524	PRP19/PSO4 homolog protein
meNOG04816	494	9606.ENSP00000304668	Histidyl-Trna synthetase
meNOG04821	493	9606.ENSP00000338113	
meNOG04826	540	9606.ENSP00000313854	Cisplatin resistance related protein CRR9p
meNOG04827	478	9606.ENSP00000352522	
meNOG04836	712	9606.ENSP00000257129	
meNOG04861	903	9606.ENSP00000261741	Rna-Binding motif protein 19
meNOG04868	401	9606.ENSP00000248924	Glycine C-acetyltransferase (2-amino-3-ketobutyrate-coenzyme A ligase)
meNOG04876	528	9606.ENSP00000304531	
meNOG04888	368	9606.ENSP00000329715	
meNOG04895	2817	9606.ENSP00000295888	
meNOG04896	484	9606.ENSP00000264279	Nucleolar protein NOP5 (Nucleolar protein 5)
meNOG04904	1812	9606.ENSP00000345834	Afadin (Protein Af-6)
meNOG04907	309	9606.ENSP00000260511	
meNOG04911	1722	9606.ENSP00000242310	
meNOG04914	372	9606.ENSP00000342562	
meNOG04917	705	9606.ENSP00000262745	Rad54-Like protein
meNOG04924	730	9606.ENSP00000215793	Splicing factor 3 subunit 1
meNOG04925	732	9606.ENSP00000270176	Protein kinase
meNOG04929	380	9606.ENSP00000284719	Gtp-Binding protein PTD004
meNOG04933	256	9606.ENSP00000296802	TGF beta-inducible nuclear protein 1 (L-name related LNR42)
meNOG04936	1103	9606.ENSP00000344055	
meNOG04945	515	9606.ENSP00000283109	RIO kinase 2
meNOG04946	623	9606.ENSP00000302728	Beta-Glucuronidase precursor
meNOG04956	582	9606.ENSP00000343344	
meNOG04962	550	9606.ENSP00000271965	Polypeptide N-acetylgalactosaminyltransferase 2 (EC 2.4.1.41)
meNOG04963	696	9606.ENSP00000296805	
meNOG04966	902	9606.ENSP00000265056	DNA replication licensing factor MCM2 (Minichromosome maintenance protein 2 homolog)
meNOG04969	549	9606.ENSP00000354966	
meNOG04970	564	9606.ENSP00000268043	
meNOG04973	696	9606.ENSP00000278224	Cysteinyl-Trna synthetase
meNOG04975	326	9606.ENSP00000321259	Transaldolase
meNOG04976	955	9606.ENSP00000249923	
meNOG04983	392	9606.ENSP00000262291	non supervised orthologous group
meNOG04985	378	9606.ENSP00000296411	Metap 1 (MAP 1) (Peptidase M 1)
meNOG04996	1276	9606.ENSP00000353877	5-Oxoprolinase
meNOG05000	682	9606.ENSP00000349613	SDA1 domain containing protein
meNOG05001	490	9606.ENSP00000012443	Serine/Threonine protein phosphatase 5 (EC 3.1.3.16)
meNOG05004	602	9606.ENSP00000296577	
meNOG05006	869	9606.ENSP00000336781	

meNOG05008	1301	9606.ENSP00000264951	Exonuclease
meNOG05026	699	9606.ENSP00000233190	
meNOG05031	1859	9606.ENSP00000317123	
meNOG05039	1252	9606.ENSP00000234420	
meNOG05040	382	9606.ENSP00000305480	
meNOG05044	891	9606.ENSP00000262489	
meNOG05045	826	9606.ENSP00000215840	
meNOG05046	762	9606.ENSP00000342961	Nuclear valosin-containing protein-like
meNOG05047	1606	9606.ENSP00000263857	Dna-Directed RNA polymerase I largest subunit (EC 2.7.7.6)
meNOG05048	976	9606.ENSP00000262982	
meNOG05054	2519	9606.ENSP00000300648	
meNOG05059	707	9606.ENSP00000221481	TFIIH basal transcription factor complex helicase subunit, DNA
meNOG05068	808	9606.ENSP00000259729	non supervised orthologous group
meNOG05073	704	9606.ENSP00000316638	
meNOG05082	556	9606.ENSP00000155840	Potassium voltage-gated channel subfamily KQT member 1 (Voltage-gated potassium channel subunit Kv7.1) IKs producing slow voltage-gated potassium channel
meNOG05088	543	9606.ENSP00000286788	Chaperonin containing TCP1, subunit 8 (theta)
meNOG05089	716	9606.ENSP00000233084	
meNOG05093	1139	9606.ENSP00000268124	DNA polymerase gamma subunit 1
meNOG05096	481	9606.ENSP00000243253	Protein transport protein Sec61 subunit alpha isoform
meNOG05099	912	9606.ENSP00000266079	
meNOG05101	414	9606.ENSP00000316335	Phenylalanyl-Trna synthetase, mitochondrial precursor
meNOG05102	1138	9606.ENSP00000298600	Ribosome biogenesis protein bms1
meNOG05103	763	9606.ENSP00000027474	
meNOG05107	778	9606.ENSP00000285398	TFIIH basal transcription factor complex helicase XPB subunit (EC 3.6.1.-)
meNOG05109	642	9606.ENSP00000252011	
meNOG05113	659	9606.ENSP00000238112	Cleavage and polyadenylation specificity factor
meNOG05120	1199	9606.ENSP00000241704	
meNOG05123	767	9606.ENSP00000339633	
meNOG05132	1084	9606.ENSP00000348708	Regulator of nonsense transcripts 2 protein
meNOG05136	681	9606.ENSP00000253577	Atp-Binding cassette, sub-family B, member 7, mitochondrial precursor
meNOG05140	572	9606.ENSP00000315835	Polypeptide N-acetylgalactosaminyltransferase 11 (EC 2.4.1.41)
meNOG05141	1335	9606.ENSP00000345728	
meNOG05142	874	9606.ENSP00000251166	Coronin-7 (70 kDa WD repeat tumor rejection antigen) protein
meNOG05143	1383	9606.ENSP00000339353	Cleavage and polyadenylation specificity factor 160 kDa subunit (CPSF 160 kDa subunit)
meNOG05147	1349	9606.ENSP00000156471	
meNOG05148	978	9606.ENSP00000298310	Serologically defined colon cancer antigen 1 protein
meNOG05149	1104	9606.ENSP00000211402	Valyl-Trna synthetase (EC 6.1.1.9)
meNOG05150	623	9606.ENSP00000261308	
meNOG05161	474	9606.ENSP00000249269	Mitochondrial processing peptidase beta subunit, mitochondrial precursor
meNOG05162	1499	9606.ENSP00000259146	
meNOG05166	788	9606.ENSP00000318147	
meNOG05179	730	9606.ENSP00000263187	
meNOG05184	1350	9606.ENSP00000273612	
meNOG05187	916	9606.ENSP00000262414	
meNOG05196	896	9606.ENSP00000309477	Iron-Responsive element binding protein 1
meNOG05199	732	9606.ENSP00000279431	
meNOG05206	834	9606.ENSP00000264156	
meNOG05216	811	9606.ENSP00000263035	
meNOG05219	595	9606.ENSP00000217315	Transmembrane 9 superfamily protein member 4
meNOG05220	553	9606.ENSP00000256398	Elongation protein 3 homolog
meNOG05224	1288	9606.ENSP00000349205	
meNOG05233	711	9606.ENSP00000323527	
meNOG05250	952	9606.ENSP00000274376	Ras GTPase-activating protein 1 (GTPase-activating protein)
meNOG05260	573	9606.ENSP00000263611	Dopamine beta hydroxylase
meNOG05275	584	9606.ENSP00000266679	
meNOG05289	655	9606.ENSP00000261600	Protein involved in mRNA transport

meNOG05297	415	9606.ENSP00000268717	
meNOG05298	1157	9606.ENSP00000335371	
meNOG05301	957	9606.ENSP00000337205	Thyroid hormone receptor-associated protein complex 100 kDa component
meNOG05303	1053	9606.ENSP00000258428	
meNOG05317	1130	9606.ENSP00000290039	
meNOG05324	623	9606.ENSP00000274457	
meNOG05341	1126	9606.ENSP00000347843	
meNOG05346	502	9606.ENSP00000301694	Nuclear prelamin A recognition factor-like
meNOG05348	819	9606.ENSP00000260946	Swi/Snf-Related matrix-associated actin-dependent regulator of chromatin subfamily A-like protein 1 (EC 3.6.1.-)
meNOG05363	1383	9606.ENSP00000262879	Gtpase activator
meNOG05367	1371	9606.ENSP00000338617	Paired amphipathic helix protein Sin3a
meNOG05369	630	9606.ENSP00000282391	
meNOG05370	1357	9606.ENSP00000313885	
meNOG05374	891	9606.ENSP00000272793	
meNOG05377	815	9606.ENSP00000189978	
meNOG05394	4762	9606.ENSP00000349892	Myc-Binding protein 2
meNOG05396	1010	9606.ENSP00000341189	Focal adhesion kinase 1 EC
meNOG05398	718	9606.ENSP00000354509	Autophagy protein
meNOG05410	632	9606.ENSP00000334868	Huntingtin-Interacting protein 14 (Huntingtin-interacting protein 3)
meNOG05418	754	9606.ENSP00000066544	Cell division cycle 27 homolog protein
meNOG05431	474	9606.ENSP00000287878	5'-Amp-Activated protein kinase subunit gamma-2 (AMPK gamma-2 chain)
meNOG05434	578	9606.ENSP00000238146	
meNOG05443	357	9606.ENSP00000345902	
meNOG05448	344	9606.ENSP00000229214	HIV-1 Rev binding protein 2
meNOG05450	1576	9606.ENSP00000319766	Atp-Binding cassette, sub-family A (ABC1), member protein
meNOG05451	568	9606.ENSP00000319457	Trna(Guanine-26,N-N) methyltransferase G26dimethyltransferase
meNOG05456	714	9606.ENSP00000245157	
meNOG05467	954	9606.ENSP00000263208	HIRA protein (TUP1-like enhancer of split protein 1)
meNOG05469	407	9606.ENSP00000346142	Udp-N-Acetylglucosamine-Dolichyl-Phosphate N-acetylglucosaminephosphotransferase
meNOG05471	462	9606.ENSP00000278916	
meNOG05478	980	9606.ENSP00000333932	Tubulin-Specific chaperone d protein
meNOG05482	610	9606.ENSP00000315700	Chromatin assembly factor 1, subunit B
meNOG05484	449	9606.ENSP00000326322	Protein involved in pentose and glucuronate interconversions
meNOG05490	415	9606.ENSP00000344868	Protein involved in cell division
meNOG05492	820	9606.ENSP00000348714	Ubiquitin-Protein ligase
meNOG05500	595	9606.ENSP00000255468	
meNOG05505	497	9606.ENSP00000275076	Glutaminyl-Trna synthase (glutamine-hydrolyzing)-like 1
meNOG05513	444	9606.ENSP00000216554	Eukaryotic translation initiation factor 5
meNOG05514	317	9606.ENSP00000256383	Eukaryotic translation initiation factor 2 subunit 1 Eukaryotic translation initiation factor 2
meNOG05517	391	9606.ENSP00000355011	Interleukin enhancer binding factor 2
meNOG05522	524	9606.ENSP00000329586	
meNOG05524	937	9606.ENSP00000330113	Box polypeptide 36
meNOG05526	864	9606.ENSP00000345877	Origin recognition complex, subunit 1-like
meNOG05534	579	9606.ENSP00000252136	
meNOG05535	2014	9606.ENSP00000285968	Porin protein
meNOG05536	587	9606.ENSP00000261206	
meNOG05539	311	9606.ENSP00000354908	
meNOG05543	364	9606.ENSP00000217117	RAE1 RNA export 1 homolog protein
meNOG05545	1033	9606.ENSP00000271640	
meNOG05548	835	9606.ENSP00000339881	
meNOG05553	881	9606.ENSP00000251143	
meNOG05559	1050	9606.ENSP00000202556	Protein phosphatase 1, regulatory
meNOG05563	395	9606.ENSP00000344201	
meNOG05564	336	9606.ENSP00000310873	Prenyltransferase
meNOG05571	1299	9606.ENSP00000231498	
meNOG05573	436	9606.ENSP00000216279	Protein involved in regulation of cyclin-dependent protein kinase

meNOG05575	629		Signal recognition particle receptor alpha subunit
meNOG05581	1380	9606.ENSP00000352070	
meNOG05585	563	9606.ENSP00000265963	TFIIH basal transcription factor complex p62 subunit
meNOG05591	477	9606.ENSP00000343488	Glutamate-Trna ligase
meNOG05593	2205	9606.ENSP00000264142	Translocated promoter region (to activated MET oncogene) protein
meNOG05606	850	9606.ENSP00000249373	Smoothened homolog precursor (SMO)
meNOG05608	643	9606.ENSP00000325863	Meiotic recombination 11 homolog A protein
meNOG05610	1001	9606.ENSP00000344798	Ret proto-oncogene protein
meNOG05616	385	9606.ENSP00000016171	
meNOG05625	601	9606.ENSP00000312066	
meNOG05633	656	9606.ENSP00000331043	
meNOG05635	339	9606.ENSP00000271047	GPI transamidase (Phosphatidylinositol-glycan biosynthesis class K protein)
meNOG05647	411	9606.ENSP00000238477	
meNOG05648	281	9606.ENSP00000319027	
meNOG05649	996	9606.ENSP00000344223	
meNOG05657	457	9606.ENSP00000293195	Nadph:Adrenodoxin oxidoreductase, mitochondrial precursor (EC 1.18.1.2) Ferredoxin
meNOG05666	680	9606.ENSP00000256216	
meNOG05668	1133	9606.ENSP00000318016	
meNOG05672	389	9606.ENSP00000288680	
meNOG05676	304	9606.ENSP00000336566	Sec13-Related protein
meNOG05679	1427	9606.ENSP00000265125	Mitogen-Activated protein kinase kinase kinase 4 (EC 2.7.11.25)
meNOG05680	573	9606.ENSP00000209827	
meNOG05683	510	9606.ENSP00000275820	RNA binding protein
meNOG05689	304	9606.ENSP00000312769	E (cyclophilin E) protein
meNOG05695	485	9606.ENSP00000261601	
meNOG05700	279	9606.ENSP00000341885	40S ribosomal protein S2
meNOG05701	604	9606.ENSP00000320885	
meNOG05702	680	9606.ENSP00000252072	Phosphorylated CTD interacting factor
meNOG05704	584	9606.ENSP00000302929	Dynein intermediate chain 2, axonemal protein
meNOG05707	753	9606.ENSP00000350341	non supervised orthologous group
meNOG05717	621	9606.ENSP00000254719	Replication protein A 70 kDa DNA-binding subunit (RP-A)
meNOG05720	486	9606.ENSP00000299626	
meNOG05727	391	9606.ENSP00000261015	WD repeat domain 12 protein
meNOG05732	1401	9606.ENSP00000349959	non supervised orthologous group
meNOG05734	390	9606.ENSP00000266254	
meNOG05739	507	9606.ENSP00000264515	Retinoblastoma binding protein 5
meNOG05742	319	9606.ENSP00000284670	Budding uninhibited by benzimidazoles 3 homolog protein
meNOG05745	501	9606.ENSP00000264639	
meNOG05749	417	9606.ENSP00000324856	
meNOG05750	620	9606.ENSP00000260373	
meNOG05758	824	9606.ENSP00000309457	
meNOG05760	447	9606.ENSP00000332646	
meNOG05765	304	9606.ENSP00000199320	
meNOG05773	273	9606.ENSP00000219252	
meNOG05782	789	9606.ENSP00000344711	Vacuolar protein sorting 16
meNOG05788	697	9606.ENSP00000257491	MAK10 homolog, amino-acid N-acetyltransferase subunit
meNOG05799	550	9606.ENSP00000261812	
meNOG05800	735	9606.ENSP00000279281	Chromosome 11 open reading frame2 protein
meNOG05805	460	9606.ENSP00000345358	Microspherule protein 1 (58 kDa microspherule protein)
meNOG05810	1606	9606.ENSP00000265562	Protein-Tyrosine-Phosphatase
meNOG05812	745	9606.ENSP00000278063	
meNOG05816	2106	9606.ENSP00000343741	Serine-Protein kinase ATR
meNOG05817	834	9606.ENSP00000303511	
meNOG05818	932	9606.ENSP00000318070	Myotubularin-Related protein 3 (EC 3.1.3.48)
meNOG05827	359	9606.ENSP00000265028	Dnaj (Hsp40) homolog, subfamily B, member 11 protein
meNOG05832	1281	9606.ENSP00000265565	
meNOG05833	1186	9606.ENSP00000343001	

meNOG05835	661	9606.ENSP00000342181	
meNOG05838	504	9606.ENSP00000261628	
meNOG05840	442	9606.ENSP00000251413	Tubulin gamma-1 chain (Gamma-1 tubulin) protein
meNOG05841	477	9606.ENSP00000317362	Pro-X carboxypeptidase
meNOG05850	850	9606.ENSP00000257215	Triacylglycerol lipase
meNOG05852	373	9606.ENSP00000307740	
meNOG05865	406	9606.ENSP00000242592	Acyl-Coa dehydrogenase, short-chain specific, mitochondrial precursor
meNOG05866	277	9606.ENSP00000264203	
meNOG05870	418	9606.ENSP00000246505	
meNOG05871	741	9606.ENSP00000308928	Probable ubiquitin carboxyl-terminal hydrolase CYLD (EC 3.1.2.15) Ubiquitin
meNOG05874	846	9606.ENSP00000354061	
meNOG05878	585	9606.ENSP00000291775	G-Protein signalling modulator
meNOG05879	487	9606.ENSP00000313311	Dnaj homolog subfamily C member 7 (Tetratricopeptide repeat protein 2)
meNOG05881	924	9606.ENSP00000272322	
meNOG05882	325	9606.ENSP00000296786	non supervised orthologous group
meNOG05883	904	9606.ENSP00000285894	La related protein
meNOG05884	354	9606.ENSP00000255484	Replication factor C (activator 1) 3
meNOG05885	608	9606.ENSP00000319169	Protein arginine N-methyltransferase
meNOG05888	1155	9606.ENSP00000261396	
meNOG05891	463	9606.ENSP00000333813	Dolichyl-P-Man:Man protein
meNOG05893	390	9606.ENSP00000295901	26S proteasome non-ATPase regulatory subunit 6 (26S proteasome regulatory subunit S10)
meNOG05896	285	9606.ENSP00000355279	CCR4-NOT transcription complex subunit 8 (CCR4-associated factor 8)
meNOG05908	566	9606.ENSP00000353910	Alpha-(1,6)-Fucosyltransferase (EC 2.4.1.68)
meNOG05909	525	9606.ENSP00000268148	De-Etiolated homolog 1 protein
meNOG05918	1747	9606.ENSP00000259335	
meNOG05924	1192	9606.ENSP00000336727	
meNOG05927	302	9606.ENSP00000317473	Rab geranylgeranyltransferase beta subunit
meNOG05950	557	9606.ENSP00000279036	GPI transamidase component PIG-T precursor
meNOG05951	317	9606.ENSP00000272402	Protein involved in positive regulation of cell proliferation
meNOG05954	372	9606.ENSP00000314228	Mitogen activated protein kinase kinase
meNOG05957	237	9606.ENSP00000278572	Ribosomal protein S3
meNOG05958	615	9606.ENSP00000348554	
meNOG05967	873	9606.ENSP00000300793	Vacuolar protein sorting 11 yeast
meNOG05972	508	9606.ENSP00000304230	
meNOG05982	833	9606.ENSP00000255305	
meNOG05985	1071	9606.ENSP00000347978	Excision repair cross-complementing rodent repair deficiency, complementation group 5 protein
meNOG05989	386	9606.ENSP00000267142	WD repeat and FYVE domain containing protein
meNOG05990	386	9606.ENSP00000262455	
meNOG05992	819	9606.ENSP00000264694	
meNOG05998	507	9606.ENSP00000216367	
meNOG06002	1305	9606.ENSP00000283351	Protein involved in transport
meNOG06003	719	9606.ENSP00000350967	
meNOG06007	328	9606.ENSP00000256761	Ubiquitin C-terminal hydrolase UCH37
meNOG06008	334	9606.ENSP00000025399	Serine-Threonine kinase receptor-associated protein (UNR-interacting protein)
meNOG06013	769	9606.ENSP00000314004	
meNOG06018	1051	9606.ENSP00000318641	non supervised orthologous group
meNOG06021	398	9606.ENSP00000235407	Cystathionine gamma-lyase (EC 4.4.1.1)
meNOG06024	690	9606.ENSP00000236256	
meNOG06030	476	9606.ENSP00000299229	
meNOG06031	779	9606.ENSP00000343666	
meNOG06033	470	9606.ENSP00000296595	non supervised orthologous group
meNOG06045	335	9606.ENSP00000040663	Translation regulator protein
meNOG06050	643	9606.ENSP00000264424	Guanylate cyclase soluble subunit beta-1 (EC 4.6.1.2)
meNOG06055	445	9606.ENSP00000262395	Tnf receptor-associated factor 4
meNOG06062	1166	9606.ENSP00000251334	non supervised orthologous group
meNOG06063	297	9606.ENSP00000313819	Ribosomal protein L5

meNOG06064	1785	9606.ENSP00000346566	
meNOG06078	843	9606.ENSP00000259455	Gamma-Aminobutyric acid type B receptor, subunit 2 precursor (GABA-B receptor 2)
meNOG06082	622	9606.ENSP00000269221	
meNOG06090	921	9606.ENSP00000352254	
meNOG06099	858	9606.ENSP00000310520	DNA repair endonuclease XPF (EC 3.1.-.-)
meNOG06100	649	9606.ENSP00000265614	
meNOG06123	629	9606.ENSP00000083182	Protein-Binding protein 2; amyloid beta precursor protein
meNOG06127	381	9606.ENSP00000305602	Serologically defined colon cancer antigen 10 protein
meNOG06138	367	9606.ENSP00000188312	
meNOG06139	432	9606.ENSP00000270538	Import inner membrane translocase subunit TIM44, mitochondrial
meNOG06141	382	9606.ENSP00000343032	
meNOG06144	860	9606.ENSP00000310449	DNA ligase IV
meNOG06159	511	9606.ENSP00000348234	
meNOG06162	481	9606.ENSP00000327592	Ubiquitin-Protein ligase
meNOG06165	1272	9606.ENSP00000297933	
meNOG06175	905	9606.ENSP00000328992	
meNOG06196	360	9606.ENSP00000296469	non supervised orthologous group
meNOG06198	1386	9606.ENSP00000354977	Zinc ion binding protein
meNOG06200	386	9606.ENSP00000354157	Sterile alpha motif domain containing 8 protein
meNOG06211	1051	9606.ENSP00000276826	
meNOG06244	961	9606.ENSP00000302239	Ubiquitin carboxyl-terminal hydrolase 8 (EC 3.1.2.15)
meNOG06245	842	9606.ENSP00000315674	Protein involved in rRNA processing
meNOG06250	1364	9606.ENSP00000351811	
meNOG06254	1803	9606.ENSP00000318295	
meNOG06267	453	9606.ENSP00000277632	Calcium ion binding protein
meNOG06271	524	9606.ENSP00000330039	
meNOG06284	503	9606.ENSP00000234393	non supervised orthologous group
meNOG06287	411	9606.ENSP00000338788	Zinc ion binding protein
meNOG06288	562	9606.ENSP00000305045	PX domain containing serine/threonine kinase
meNOG06299	262	9606.ENSP00000044462	Proteasome component C9 (Macropain subunit C9)
meNOG06301	321	9606.ENSP00000301724	G protein beta subunit
meNOG06302	769	9606.ENSP00000266123	Muts protein homolog
meNOG06325	483	9606.ENSP00000261250	non supervised orthologous group
meNOG06327	425	9606.ENSP00000001008	
meNOG06334	826	9606.ENSP00000248125	
meNOG06336	500	9606.ENSP00000264883	
meNOG06341	571	9606.ENSP00000319918	
meNOG06350	546	9606.ENSP00000244230	M-Phase phosphoprotein 10 (U3 small nucleolar ribonucleoprotein)
meNOG06354	944	9606.ENSP00000267229	
meNOG06357	702	9606.ENSP00000322807	
meNOG06359	955	9606.ENSP00000319149	non supervised orthologous group
meNOG06362	481	9606.ENSP00000256579	
meNOG06378	1056	9606.ENSP00000248633	Peroxisome biogenesis factor 1
meNOG06390	479	9606.ENSP00000304233	
meNOG06391	508	9606.ENSP00000341796	Mitogen activated protein kinase kinase kinase 7
meNOG06399	369	9606.ENSP00000289382	non supervised orthologous group
meNOG06412	629	9606.ENSP00000245544	
meNOG06415	829	9606.ENSP00000228284	
meNOG06428	665	9606.ENSP00000320599	
meNOG06437	413	9606.ENSP00000264108	
meNOG06438	470	9606.ENSP00000263167	D-3-Phosphoglycerate dehydrogenase (EC 1.1.1.95)
meNOG06445	378	9606.ENSP00000321591	
meNOG06456	371	9606.ENSP00000247138	Udp-Galactose translocator (UDP-galactose transporter) protein
meNOG06479	440	9606.ENSP00000341805	
meNOG06483	282	9606.ENSP00000346067	40S ribosomal protein SA (p40)
meNOG06509	593	9606.ENSP00000163416	
meNOG06518	518	9606.ENSP00000296473	Protein involved in establishment of localization

meNOG06541	880	9606.ENSP00000311750	non supervised orthologous group
meNOG06550	786	9606.ENSP00000281172	Epidermal growth factor receptor kinase substrate
meNOG06558	348	9606.ENSP00000295240	
meNOG06578	444	9606.ENSP00000297431	
meNOG06585	998	9606.ENSP00000345782	
meNOG06593	497	9606.ENSP00000345766	Translation initiation factor eIF-2B delta subunit
meNOG06616	447	9606.ENSP00000281038	
meNOG06620	451	9606.ENSP00000310042	
meNOG06628	694	9606.ENSP00000282251	
meNOG06629	304	9606.ENSP00000215567	Synaptic glycoprotein SC2
meNOG06631	336	9606.ENSP00000266126	Translation initiation factor eIF-2B subunit beta (eIF-2B GDP-GTP exchange factor subunit beta)
meNOG06633	913	9606.ENSP00000300917	
meNOG06639	534	9606.ENSP00000229971	F-Box and leucine-rich repeat protein 4
meNOG06643	523	9606.ENSP00000319369	non supervised orthologous group
meNOG06652	653	9606.ENSP00000225696	Nuclear pore complex protein Nup88 (Nucleoporin Nup88)
meNOG06659	391	9606.ENSP00000263733	
meNOG06670	447	9606.ENSP00000232888	U3 small nucleolar RNA-interacting protein 2 U3 small nucleolar
meNOG06672	951	9606.ENSP00000323339	
meNOG06681	597	9606.ENSP00000347169	
meNOG06688	666	9606.ENSP00000305442	
meNOG06693	446	9606.ENSP00000349658	Exopeptidase
meNOG06703	473	9606.ENSP00000296792	
meNOG06704	298	9606.ENSP00000262812	Coatomer epsilon subunit; epsilon coat protein
meNOG06716	871	9606.ENSP00000349568	non supervised orthologous group
meNOG06718	338	9606.ENSP00000296273	Replication factor C (activator 1) 4
meNOG06730	484	9606.ENSP00000264689	non supervised orthologous group
meNOG06733	943	9606.ENSP00000353793	
meNOG06734	905	9606.ENSP00000204604	Protein involved in development
meNOG06737	488	9606.ENSP00000185150	
meNOG06753	1109	9606.ENSP00000264106	Integrin alpha-6 precursor (VLA-6) (CD49f)
meNOG06761	609	9606.ENSP00000270460	EPS-15 interacting protein
meNOG06772	439	9606.ENSP00000231749	Zinc finger MYND domain-containing protein 10
meNOG06780	578	9606.ENSP00000019317	Rala-Binding protein 1 (RalBP1)
meNOG06785	954	9606.ENSP00000295709	Serine/Threonine kinase 36
meNOG06791	222	9606.ENSP00000238339	
meNOG06800	701	9606.ENSP00000353564	Protein involved in transport
meNOG06831	332	9606.ENSP00000263716	
meNOG06849	946	9606.ENSP00000295760	Calcium ion binding protein
meNOG06870	353	9606.ENSP00000301329	Lactoylglutathione lyase
meNOG06872	567	9606.ENSP00000265774	RNA binding protein
meNOG06879	543	9606.ENSP00000257347	Protein involved in cysteinyl-tRNA aminoacylation
meNOG06889	295	9606.ENSP00000025429	Deoxyribose-Phosphate aldolase
meNOG06894	501	9606.ENSP00000327483	Golgi resident protein GCP60 (Acyl-CoA binding domain containing protein 3)
meNOG06902	425	9606.ENSP00000002125	non supervised orthologous group
meNOG06903	466	9606.ENSP00000262288	Serine carboxypeptidase 1
meNOG06904	353	9606.ENSP00000296642	
meNOG06906	324	9606.ENSP00000348477	Solute carrier family 39 (metal ion transporter), member 11 protein
meNOG06922	662	9606.ENSP00000223073	RNA binding protein
meNOG06925	364	9606.ENSP00000296484	non supervised orthologous group
meNOG06929	483	9606.ENSP00000268057	
meNOG06932	390	9606.ENSP00000219240	
meNOG06935	287	9606.ENSP00000235287	Spermidine synthase
meNOG06945	755	9606.ENSP00000260382	
meNOG06953	459	9606.ENSP00000315671	non supervised orthologous group
meNOG06969	343	9606.ENSP00000272801	
meNOG06974	435	9606.ENSP00000283875	Transcription initiation factor IIE alpha subunit (TFIIE-alpha)
meNOG06975	415	9606.ENSP00000264151	

meNOG06997	460	9606.ENSP00000286070	Developmentally regulated RNA binding protein 1
meNOG07008	235	9606.ENSP00000320413	
meNOG07010	361	9606.ENSP00000240802	RING finger protein 2 (RING finger protein 1B)
meNOG07012	523	9606.ENSP00000265465	DNA polymerase alpha 70 kDa subunit
meNOG07023	348	9606.ENSP00000216910	
meNOG07047	491	9606.ENSP00000258531	
meNOG07052	353	9606.ENSP00000345341	Prostaglandin E synthase 2 (EC 5.3.99.3)
meNOG07064	419	9606.ENSP00000303570	Zinc ion binding protein
meNOG07066	318	9606.ENSP00000233741	
meNOG07079	250	9606.ENSP00000261700	
meNOG07090	405	9606.ENSP00000354501	Metal ion binding protein
meNOG07093	438	9606.ENSP00000265287	Oxidoreductase
meNOG07099	554	9606.ENSP00000260210	
meNOG07109	261	9606.ENSP00000314733	Toll interacting protein
meNOG07111	682	9606.ENSP00000338018	Hypoxia-Inducible factor 1 alpha (HIF-1 alpha)
meNOG07112	360	9606.ENSP00000259365	Tropomodulin 1 protein
meNOG07130	244	9606.ENSP00000327268	EC 1.6.5.3 protein
meNOG07138	761	9606.ENSP00000288199	
meNOG07140	473	9606.ENSP00000327546	non supervised orthologous group
meNOG07143	566	9606.ENSP00000259259	
meNOG07158	364	9606.ENSP00000350792	Protein involved in chromosome organization and biogenesis
meNOG07172	297	9606.ENSP00000292035	Cofactor required for Sp1 transcriptional activation, subunit 8
meNOG07192	518	9606.ENSP00000327436	non supervised orthologous group
meNOG07193	324	9606.ENSP00000290429	
meNOG07198	528	9606.ENSP00000349898	
meNOG07200	533	9606.ENSP00000309430	GPI transamidase component PIG-S
meNOG07244	279	9606.ENSP00000220853	non supervised orthologous group
meNOG07254	419	9606.ENSP00000340029	Zinc finger protein
meNOG07289	466	9606.ENSP00000326968	
meNOG07303	284	9606.ENSP00000244689	Glycine N-methyltransferase
meNOG07314	480	9606.ENSP00000252137	DGCR14 protein DiGeorge syndrome critical region 14
meNOG07349	371	9606.ENSP00000217326	
meNOG07353	636	9606.ENSP00000280700	
meNOG07360	466	9606.ENSP00000252856	Ubiquitin associated domain containing 1 protein
meNOG07362	297	9606.ENSP00000264020	non supervised orthologous group
meNOG07372	391	9606.ENSP00000281273	
meNOG07387	660	9606.ENSP00000348512	
meNOG07396	854	9606.ENSP00000340298	Zinc ion binding protein
meNOG07425	289	9606.ENSP00000277884	
meNOG07433	953	9606.ENSP00000348582	
meNOG07438	327	9606.ENSP00000230354	Tata-Box binding protein (TATA-box factor)
meNOG07455	313	9606.ENSP00000315152	
meNOG07459	348	9606.ENSP00000235521	
meNOG07460	241	9606.ENSP00000354744	Adenylate kinase isoenzyme 2, mitochondrial
meNOG07467	558	9606.ENSP00000338727	
meNOG07475	248	9606.ENSP00000304601	Hepatocellular carcinoma-associated antigen 127 protein
meNOG07486	559	9606.ENSP00000299213	Protein involved in RNA export from nucleus
meNOG07496	381	9606.ENSP00000272748	non supervised orthologous group
meNOG07498	286	9606.ENSP00000250937	
meNOG07524	234	9606.ENSP00000262193	Proteasome subunit beta type 1 (EC 3.4.25.1) Proteasome
meNOG07556	383	9606.ENSP00000232501	Tumor suppressor candidate 4 protein
meNOG07569	487	9606.ENSP00000209873	Achalasia, adrenocortical insufficiency, alacrimia protein
meNOG07571	465	9606.ENSP00000327545	Opsonin receptor
meNOG07581	388	9606.ENSP00000308022	
meNOG07597	280	9606.ENSP00000007264	Pseudouridine synthase
meNOG07614	443	9606.ENSP00000247026	
meNOG07630	391	9606.ENSP00000246044	Bone morphogenetic protein 2
meNOG07634	308	9606.ENSP00000248069	Solute carrier family 35, member protein

meNOG07648	268	9606.ENSP00000263774	Nadh-Ubiquinone oxidoreductase 30 kDa subunit, mitochondrial precursor
meNOG07650	430	9606.ENSP00000339293	Nin one binding protein
meNOG07653	469	9606.ENSP00000261249	Methyltransferase
meNOG07673	321	9606.ENSP00000313446	non supervised orthologous group
meNOG07692	872	9606.ENSP00000170564	
meNOG07700	579	9606.ENSP00000350725	
meNOG07730	303	9606.ENSP00000277359	Surfeit locus protein
meNOG07738	474	9606.ENSP00000261407	
meNOG07754	428	9606.ENSP00000265650	non supervised orthologous group
meNOG07770	334	9606.ENSP00000270225	Ubiquitin-Like 1 activating enzyme E1A (SUMO-1 activating enzyme subunit 1)
meNOG07800	369	9606.ENSP00000333346	
meNOG07832	209	9606.ENSP00000300291	Cleavage and polyadenylation specific factor 5, 25
meNOG07837	334	9606.ENSP00000319255	Cysteine and histidine-rich domain (CHORD)-containing, zinc-binding protein
meNOG07846	337	9606.ENSP00000343499	Deaminase
meNOG07851	248	9606.ENSP00000168216	3-Hydroxyacyl-Coa dehydrogenase type-2 (EC 1.1.1.35)
meNOG07868	279	9606.ENSP00000321330	Ubiquitin conjugating enzyme 7 interacting protein 4
meNOG07871	318	9606.ENSP00000261888	NAD+ ADP-ribosyltransferase
meNOG07877	282	9606.ENSP00000301920	non supervised orthologous group
meNOG07888	299	9606.ENSP00000299201	Methyltransferase
meNOG07912	382	9606.ENSP00000212369	SH3 domain GRB2-like protein B1 (EC 2.3.1.-)
meNOG07920	409	9606.ENSP00000311144	WD40 repeats
meNOG07928	505	9606.ENSP00000269346	non supervised orthologous group
meNOG07946	460	9606.ENSP00000287996	
meNOG07953	406	9606.ENSP00000013070	
meNOG07965	446	9606.ENSP00000284973	
meNOG07972	392	9606.ENSP00000266517	Ethanolamine kinase (EC 2.7.1.82)
meNOG08007	400	9606.ENSP00000219345	1-O-Acylceramide synthase precursor (EC 2.3.1.-)
meNOG08010	371	9606.ENSP00000311535	Cisplatin resistance-associated overexpressed protein
meNOG08025	282	9606.ENSP00000348168	
meNOG08053	276	9606.ENSP00000229447	Protein involved in electron transport
meNOG08056	392	9606.ENSP00000303129	
meNOG08065	395	9606.ENSP00000334140	
meNOG08086	890	9606.ENSP00000339958	
meNOG08090	300	9606.ENSP00000342267	Mitochondrial ornithine transporter 1 (Solute carrier family 25, member 15) protein
meNOG08101	358	9606.ENSP00000282512	
meNOG08119	367	9606.ENSP00000262213	Associating membrane protein 1
meNOG08134	274	9606.ENSP00000251159	
meNOG08138	533	9606.ENSP00000304853	
meNOG08148	336	9606.ENSP00000216489	
meNOG08205	370	9606.ENSP00000319910	
meNOG08223	847	9606.ENSP00000348550	FERM domain-containing protein
meNOG08231	554	9606.ENSP00000324693	Protein involved in regulation of tracheal tube architecture
meNOG08247	727	9606.ENSP00000258255	
meNOG08257	1294	9606.ENSP00000267974	Nuclear receptor coactivator 2 (NCoA-2)
meNOG08312	375	9606.ENSP00000322180	non supervised orthologous group
meNOG08331	362	9606.ENSP00000216879	NSFL1 cofactor p47 (p97 cofactor p47)
meNOG08380	1084	9606.ENSP00000337250	Additional sex combs like 2 protein
meNOG08381	329	9606.ENSP00000303366	Vesicular integral-membrane protein VIP36 precursor (Lectin, mannose-binding 2)
meNOG08384	292	9606.ENSP00000259342	non supervised orthologous group
meNOG08402	231	9606.ENSP00000278359	Protein involved in apoptosis
meNOG08410	665	9606.ENSP00000234697	Transcription elongation factor B polypeptide 3 (RNA polymerase II transcription factor SIII subunit A)
meNOG08416	401	9606.ENSP00000253003	Adhesion-Regulating molecule 1 precursor (110 kDa cell membrane glycoprotein)
meNOG08417	652	9606.ENSP00000260926	Special AT-rich sequence binding protein 2
meNOG08430	361	9606.ENSP00000295982	Cysteine-Rich with EGF-like domains 1 protein
meNOG08486	344	9606.ENSP00000216479	AHA1, activator of heat shock 90kDa protein ATPase homolog 1

meNOG08500	303	9606.ENSP00000261247	non supervised orthologous group
meNOG08508	216	9606.ENSP00000339193	
meNOG08521	536	9606.ENSP00000345699	Protein involved in regulation of cellular process
meNOG08546	693	9606.ENSP00000196169	Tudor domain containing protein
meNOG08549	470	9606.ENSP00000225402	
meNOG08555	429	9606.ENSP00000320566	Inhibitor of growth family, member protein
meNOG08573	1143	9606.ENSP00000306983	Protein involved in regulation of transcription, DNA-dependent
meNOG08582	344	9606.ENSP00000313318	
meNOG08584	501	9606.ENSP00000164305	
meNOG08587	276	9606.ENSP00000312792	
meNOG08590	211	9606.ENSP00000351896	
meNOG08600	585	9606.ENSP00000334095	Nf-Kappa-B-Repressing factor
meNOG08609	712	9606.ENSP00000346144	non supervised orthologous group
meNOG08620	983	9606.ENSP00000205214	
meNOG08626	328	9606.ENSP00000295119	
meNOG08631	486	9606.ENSP00000265960	
meNOG08639	407	9606.ENSP00000277540	WD40 repeats
meNOG08674	242	9606.ENSP00000225724	
meNOG08679	427	9606.ENSP00000310491	Rho-Gtpase-Activating protein 1
meNOG08713	686	9606.ENSP00000352655	Metal ion transporter protein
meNOG08729	815	9606.ENSP00000301280	Chromatin assembly factor 1, subunit A
meNOG08746	987	9606.ENSP00000344087	Autism susceptibility gene 2 protein
meNOG08747	419	9606.ENSP00000271552	Phosphatidylinositol glycan, class M protein
meNOG08752	207	9606.ENSP00000341730	60S ribosomal protein L10 (QM protein homolog)
meNOG08762	280	9606.ENSP00000187762	non supervised orthologous group
meNOG08826	580	9606.ENSP00000262313	Gene telomeric to alpha globin cluster protein
meNOG08857	716	9606.ENSP00000264645	Protein CASC3 (Cancer susceptibility candidate gene 3 protein)
meNOG08861	278	9606.ENSP00000343100	
meNOG08867	274	9606.ENSP00000225000	Thioredoxin-Like protein 2 (PKC-interacting cousin of thioredoxin)
meNOG08868	307	9606.ENSP00000317687	Inorganic pyrophosphatase (EC 3.6.1.1)
meNOG08922	589	9606.ENSP00000329841	non supervised orthologous group
meNOG08934	261	9606.ENSP00000249442	Metaxin 2 protein
meNOG08941	298	9606.ENSP00000228955	General transcription factor iih polypeptide 3
meNOG08952	727	9606.ENSP00000240423	Condensin complex subunit 2 (Barren homolog protein 1)
meNOG08959	957	9606.ENSP00000261778	non supervised orthologous group
meNOG08962	397	9606.ENSP00000346785	Dynactin subunit 2 (Dynactin complex 50 kDa subunit)
meNOG08988	1992	9606.ENSP00000351894	Nuclear receptor coactivator 6 (Amplified in breast cancer protein 3)
meNOG08995	506	9606.ENSP00000238823	non supervised orthologous group
meNOG08996	1164	9606.ENSP00000239440	Centaurin-Delta 3 (Cnt-d3) protein
meNOG09002	504	9606.ENSP00000309960	Transmembrane protein 15
meNOG09008	1676	9606.ENSP00000259310	Porin protein
meNOG09037	378	9606.ENSP00000338287	
meNOG09048	352	9606.ENSP00000327541	Basic leucine zipper nuclear factor 1
meNOG09058	561	9606.ENSP00000275073	
meNOG09077	510	9606.ENSP00000260506	Formin binding protein 1-like
meNOG09080	659	9606.ENSP00000350063	
meNOG09085	567	9606.ENSP00000336752	
meNOG09090	965	9606.ENSP00000350579	non supervised orthologous group
meNOG09097	315	9606.ENSP00000311984	non supervised orthologous group
meNOG09102	419	9606.ENSP00000321810	
meNOG09118	312	9606.ENSP00000321850	Hormone
meNOG09133	425	9606.ENSP00000216639	Serine/Threonine-Protein kinase VRK1 (EC 2.7.11.1)
meNOG09145	399	9606.ENSP00000231526	
meNOG09150	467	9606.ENSP00000264892	Sh3-Domain binding protein 2
meNOG09189	416	9606.ENSP00000217446	
meNOG09192	450	9606.ENSP00000245615	Leukocyte receptor cluster (LRC) member 4
meNOG09194	292	9606.ENSP00000257288	TPA regulated locus protein
meNOG09253	402	9606.ENSP00000261643	COX10 homolog, cytochrome c oxidase assembly protein, heme A:

			farnesyltransferase
meNOG09307	378	9606.ENSP00000272418	Mitochondrial 28S ribosomal protein S5
meNOG09332	339	9606.ENSP00000225873	Peroxisome assembly protein 12 (Peroxin-12)
meNOG09348	1150	9606.ENSP00000223950	Ankyrin repeat domain protein
meNOG09364	465	9606.ENSP00000266848	non supervised orthologous group
meNOG09381	978	9606.ENSP00000265987	Sorbin and SH3 domain containing protein
meNOG09411	507	9606.ENSP00000310321	
meNOG09423	307	9606.ENSP00000263741	Stromal cell derived factor 4
meNOG09426	1445	9606.ENSP00000348854	Nuclear pore complex protein Nup153
meNOG09428	338	9606.ENSP00000276682	Eukaryotic translation initiation factor 3, subunit 3 (gamma)
meNOG09489	1094	9606.ENSP00000313084	
meNOG09532	422	9606.ENSP00000262366	GLIS family zinc finger 2 protein
meNOG09533	612	9606.ENSP00000299853	Dna-Directed RNA polymerases III
meNOG09559	340	9606.ENSP00000303423	Protein farnesyltransferase/geranylgeranyltransferase type I alpha subunit (EC 2.5.1.58)
meNOG09585	490	9606.ENSP00000322716	
meNOG09607	242	9606.ENSP00000324153	N-Acetyltransferase
meNOG09610	330	9606.ENSP00000337476	non supervised orthologous group
meNOG09633	499	9606.ENSP00000269932	non supervised orthologous group
meNOG09643	447	9606.ENSP00000263297	non supervised orthologous group
meNOG09652	384	9606.ENSP00000254286	ARP10 actin-related protein 10 homolog
meNOG09655	295	9606.ENSP00000155926	Tribbles homolog 2 protein
meNOG09690	461	9606.ENSP00000283009	Chromatin binding protein
meNOG09693	720	9606.ENSP00000295952	Unfolded protein binding
meNOG09727	511	9606.ENSP00000275747	non supervised orthologous group
meNOG09728	314	9606.ENSP00000338030	START domain containing 7 protein
meNOG09746	1616	9606.ENSP00000352463	Transcription factor 20 (Stromelysin 1 PDGF-responsive element-binding protein)
meNOG09769	348	9606.ENSP00000260605	
meNOG09786	407	9606.ENSP00000262633	RNA binding protein
meNOG09790	654	9606.ENSP00000338387	Protein ligase
meNOG09794	294	9606.ENSP00000253546	
meNOG09807	310	9606.ENSP00000340034	
meNOG09809	313	9606.ENSP00000273037	
meNOG09866	514	9606.ENSP00000286353	Acid phosphatase
meNOG09870	577	9606.ENSP00000293970	Protein involved in cell development
meNOG09881	332	9606.ENSP00000031512	
meNOG09925	248	9606.ENSP00000264452	
meNOG09926	293	9606.ENSP00000260102	
meNOG09932	732	9606.ENSP00000251038	
meNOG09933	328	9606.ENSP00000251152	Sin3 histone deacetylase corepressor complex component
meNOG10015	309	9606.ENSP00000315680	Peroxisome targeting signal-2 binding protein
meNOG10022	331	9606.ENSP00000266025	non supervised orthologous group
meNOG10041	534	9606.ENSP00000163059	
meNOG10056	218	9606.ENSP00000355258	Ribosomal protein L10a
meNOG10064	276	9606.ENSP00000334401	
meNOG10076	273	9606.ENSP00000329624	Protein involved in cytoskeleton organization and biogenesis
meNOG10088	269	9606.ENSP00000239893	
meNOG10089	313	9606.ENSP00000239891	
meNOG10092	218	9606.ENSP00000338990	non supervised orthologous group
meNOG10094	501	9606.ENSP00000312411	Calmodulin-Dependent protein kinase phosphatase
meNOG10099	351	9606.ENSP00000258455	
meNOG10106	377	9606.ENSP00000263884	
meNOG10125	301	9606.ENSP00000217225	Chromosome 20 open reading frame 7 isoform 1 protein
meNOG10128	633	9606.ENSP00000264344	non supervised orthologous group
meNOG10137	309	9606.ENSP00000307087	
meNOG10141	619	9606.ENSP00000341947	non supervised orthologous group
meNOG10163	230	9606.ENSP00000280734	non supervised orthologous group
meNOG10167	302	9606.ENSP00000354445	

meNOG10172	587	9606.ENSP00000296288	Ubiquitin C-terminal hydrolase X4
meNOG10178	489	9606.ENSP00000277554	Protein binding
meNOG10193	934	9606.ENSP00000283645	Protein involved in microtubule-based process
meNOG10196	385	9606.ENSP00000294374	Tetratricopeptide repeat
meNOG10201	254	9606.ENSP00000216455	Proteasome component C8 (Macropain subunit C8)
meNOG10231	905	9606.ENSP00000352668	Rna-Binding motif protein 12
meNOG10314	1452	9606.ENSP00000264555	Protein involved in transcription, DNA-dependent
meNOG10316	320	9606.ENSP00000345931	Calcium ion binding protein
meNOG10330	924	9606.ENSP00000259161	Phosphoinositide binding protein
meNOG10355	233	9606.ENSP00000353246	Mak16-Like protein RBM13 (RNA binding motif protein 13)
meNOG10388	264	9606.ENSP00000308533	
meNOG10429	344	9606.ENSP00000338862	
meNOG10626	309	9606.ENSP00000298248	
meNOG10645	453	9606.ENSP00000333946	
meNOG10672	319	9606.ENSP00000277749	
meNOG10673	303	9606.ENSP00000259526	Nephroblastoma overexpressed gene protein
meNOG10719	347	9606.ENSP00000323733	Protein involved in regulation of biological process
meNOG10728	433	9606.ENSP00000194006	non supervised orthologous group
meNOG10730	256	9606.ENSP00000288855	Low density lipoprotein receptor adapter protein 1 Autosomal recessive hypercholesterolemia protein
meNOG10739	254	9606.ENSP00000328216	non supervised orthologous group
meNOG10797	277	9606.ENSP00000346300	SH3/SH2 adaptor protein
meNOG10832	291	9606.ENSP00000279552	DNA binding protein
meNOG10858	645	9606.ENSP00000253699	FYVE finger-containing Rab5 effector protein rabenosyn-5
meNOG10925	566	9606.ENSP00000198767	RRN3 RNA polymerase I transcription factor homolog
meNOG10933	274	9606.ENSP00000335416	Acyltransferase
meNOG10974	286	9606.ENSP00000319739	
meNOG10985	346	9606.ENSP00000339404	Activating signal cointegrator 1 complex subunit 1
meNOG10993	704	9606.ENSP00000324771	Sh3-Domain kinase binding protein 1
meNOG11045	460	9606.ENSP00000349096	Interleukin-1 receptor-associated kinase 4
meNOG11053	752	9606.ENSP00000197268	Protein involved in cell-matrix adhesion
meNOG11072	237	9606.ENSP00000279839	Protein involved in nuclear mRNA splicing, via spliceosome
meNOG11074	258	9606.ENSP00000315469	non supervised orthologous group
meNOG11115	262	9606.ENSP00000343559	
meNOG11157	567	9606.ENSP00000305711	Cytoskeletal protein binding
meNOG11207	351	9606.ENSP00000167218	Programmed cell death protein 2 (Zinc finger protein Rp-8)
meNOG11211	287	9606.ENSP00000263177	Phosphatidate phosphatase
meNOG11215	288	9606.ENSP00000163678	S-Adenosylmethionine-Dependent methyltransferase
meNOG11232	403	9606.ENSP00000266427	Ets variant gene 6 protein
meNOG11243	183	9606.ENSP00000302896	40S ribosomal protein
meNOG11292	494	9606.ENSP00000302913	
meNOG11314	277	9606.ENSP00000290663	
meNOG11322	482	9606.ENSP00000263028	Cleavage stimulation factor, 64 kDa subunit
meNOG11330	410	9606.ENSP00000324517	non supervised orthologous group
meNOG11353	248	9606.ENSP00000246868	Shwachman-Bodian-Diamond syndrome protein
meNOG11370	955	9606.ENSP00000352510	non supervised orthologous group
meNOG11385	256	9606.ENSP00000319964	Peroxiredoxin-4 (EC 1.11.1.15) (Prx-IV) protein
meNOG11400	550	9606.ENSP00000239690	
meNOG11437	431	9606.ENSP00000261942	
meNOG11453	348	9606.ENSP00000313278	Heat shock protein binding
meNOG11463	554	9606.ENSP00000301019	DNA replication factor Cdt1 (Double parked homolog)
meNOG11491	382	9606.ENSP00000303992	non supervised orthologous group
meNOG11498	514	9606.ENSP00000350937	Zinc ion binding protein
meNOG11557	322	9606.ENSP00000288774	Peroxisome assembly protein 10
meNOG11559	271	9606.ENSP00000219548	STIP1 homology and U-box containing protein 1
meNOG11566	272	9606.ENSP00000304017	
meNOG11581	472	9606.ENSP00000258621	
meNOG11609	348	9606.ENSP00000267103	MYG1 protein

meNOG11671	438	9606.ENSP00000314615	Adp-Ribosylation factor 1 GTPase activating protein
meNOG11760	398	9606.ENSP00000248450	
meNOG11791	249	9606.ENSP00000343757	non supervised orthologous group
meNOG11792	848	9606.ENSP00000316426	Myeloid/Lymphoid or mixed lineage-leukemia protein
meNOG11828	270	9606.ENSP00000323387	Ankyrin repeat domain 29 protein
meNOG11836	372	9606.ENSP00000325423	Inositol polyphosphate-1-phosphatase
meNOG11860	655	9606.ENSP00000318977	Nuclease
meNOG11866	322	9606.ENSP00000263449	non supervised orthologous group
meNOG11875	279	9606.ENSP00000258774	Protein involved in response to DNA damage stimulus
meNOG11878	599	9606.ENSP00000267546	
meNOG11908	339	9606.ENSP00000305810	
meNOG11969	441		non supervised orthologous group
meNOG11970	307	9606.ENSP00000350290	
meNOG11982	428	9606.ENSP00000337298	
meNOG12079	332	9606.ENSP00000278903	Etoposide induced protein
meNOG12091	303	9606.ENSP00000263579	
meNOG12094	568	9606.ENSP00000341351	Wd-Repeat protein
meNOG12129	301	9606.ENSP00000352711	non supervised orthologous group
meNOG12156	351	9606.ENSP00000350685	Fasciculation and elongation protein zeta 2 (Zygin-2)
meNOG12185	262	9606.ENSP00000319141	Cytochrome b reductase 1
meNOG12196	291	9606.ENSP00000337448	
meNOG12242	255	9606.ENSP00000315309	Proteasome subunit alpha type 1 (EC 3.4.25.1)
meNOG12277	279	9606.ENSP00000265564	Exosome complex exonuclease RRP42
meNOG12283	623	9606.ENSP00000300682	Endo-Beta-N-Acetylglucosaminidase
meNOG12305	698	9606.ENSP00000264867	Peroxisome proliferator-activated receptor gamma coactivator 1-alpha (PPAR gamma coactivator 1-alpha)
meNOG12345	587	9606.ENSP00000323099	
meNOG12360	286	9606.ENSP00000011652	non supervised orthologous group
meNOG12403	376	9606.ENSP00000329359	
meNOG12405	260	9606.ENSP00000300605	Haloacid dehalogenase-like hydrolase domain containing 2
meNOG12425	314	9606.ENSP00000282486	
meNOG12434	363	9606.ENSP00000286096	non supervised orthologous group
meNOG12484	497	9606.ENSP00000257548	
meNOG12521	223	9606.ENSP00000236273	Gcip-Interacting protein p29
meNOG12531	301	9606.ENSP00000255631	non supervised orthologous group
meNOG12539	685	9606.ENSP00000200135	
meNOG12564	233	9606.ENSP00000259512	
meNOG12575	655	9606.ENSP00000234543	F-Box protein
meNOG12587	382	9606.ENSP00000290846	Trna 5-methylaminomethyl-2-thiouridylate methyltransferase
meNOG12725	300	9606.ENSP00000262507	
meNOG12732	323	9606.ENSP00000337332	
meNOG12746	273	9606.ENSP00000231238	
meNOG12793	330	9606.ENSP00000173569	THUMP domain containing protein
meNOG12897	228	9606.ENSP00000286317	Cofactor required for Sp1 transcriptional activation subunit 9 (Transcriptional coactivator CRSP33) ARC34
meNOG12903	178	9606.ENSP00000346340	COP9 signalosome complex subunit 8 (Signalosome subunit 8)
meNOG12905	391	9606.ENSP00000254928	
meNOG12936	553	9606.ENSP00000325290	Serine/Threonine-Protein kinase Haspin homolog EC
meNOG12965	454	9606.ENSP00000330484	RNA binding protein
meNOG12985	601	9606.ENSP00000338457	Hermansky-Pudlak syndrome 4 homolog protein
meNOG12991	268	9606.ENSP00000263891	
meNOG13009	1077	9606.ENSP00000351475	PAS domain-containing serine/threonine-protein kinase EC
meNOG13048	299	9606.ENSP00000263214	
meNOG13074	234	9606.ENSP00000254193	
meNOG13079	1107	9606.ENSP00000278935	Domain with 2 conserved Trp (W) residues
meNOG13106	223	9606.ENSP00000258416	Eukaryotic translation initiation factor 4E type 3 (eIF4E type 3)
meNOG13135	219	9606.ENSP00000262120	Twisted gastrulation homolog protein
meNOG13173	701	9606.ENSP00000294785	Protein involved in protein processing
meNOG13176	469	9606.ENSP00000203001	Protein involved in regulation of translational initiation

meNOG13178	340	9606.ENSP00000272091	
meNOG13198	236	9606.ENSP00000256545	
meNOG13217	829	9606.ENSP00000297153	MAM domain-containing glycosylphosphatidylinositol anchor protein 1 precursor
meNOG13220	218	9606.ENSP00000261263	
meNOG13388	221	9606.ENSP00000223500	Charged multivesicular body protein 5 (Chromatin-modifying protein 5)
meNOG13441	212	9606.ENSP00000346408	Kv channel interacting protein 4
meNOG13446	275	9606.ENSP00000203630	Myeloid leukemia factor 2 (Myelodysplasia-myeloid leukemia factor 2)
meNOG13478	415	9606.ENSP00000326858	5-Nucleotidase, cytosolic II-like 1
meNOG13521	207	9606.ENSP00000263856	
meNOG13544	337	9606.ENSP00000328671	Wd-Repeat protein
meNOG13580	216	9606.ENSP00000264255	Thioredoxin domain-containing protein 9
meNOG13642	281	9606.ENSP00000218652	Nedd4 family-interacting protein 2 (Nedd4 WW domain-binding protein 5A)
meNOG13646	320	9606.ENSP00000328455	Limbic system-associated membrane protein precursor
meNOG13764	240	9606.ENSP00000323469	non supervised orthologous group
meNOG13794	218	9606.ENSP00000273368	Transgelin-3 (Neuronal protein NP25)
meNOG13806	431	9606.ENSP00000337759	DOM-3 homolog Z protein
meNOG13816	286	9606.ENSP00000295890	
meNOG13931	265	9606.ENSP00000261666	28S ribosomal protein S31, mitochondrial precursor
meNOG14106	319	9606.ENSP00000237822	non supervised orthologous group
meNOG14175	325	9606.ENSP00000194039	Dual specificity protein phosphatase 12 (EC 3.1.3.48) Dual-specificity
meNOG14177	228	9606.ENSP00000303518	
meNOG14339	199	9606.ENSP00000314343	non supervised orthologous group
meNOG14356	267	9606.ENSP00000221486	Ribonuclease HI large subunit (EC 3.1.26.4)
meNOG14388	248	9606.ENSP00000312458	non supervised orthologous group
meNOG14393	302	9606.ENSP00000254759	Hexaprenyldihydroxybenzoate methyltransferase, mitochondrial precursor
meNOG14402	198	9606.ENSP00000320491	non supervised orthologous group
meNOG14417	231	9606.ENSP00000242285	Clathrin light chain A (Lca) protein
meNOG14451	283	9606.ENSP00000007390	non supervised orthologous group
meNOG14462	230	9606.ENSP00000259873	28S ribosomal protein S18b, mitochondrial precursor
meNOG14491	456	9606.ENSP00000330219	Synapsin III protein
meNOG14609	379	9606.ENSP00000254605	Methyltransferase
meNOG14623	346	9606.ENSP00000239880	WW domain-binding protein 4 (WBP-4)
meNOG14724	249	9606.ENSP00000323046	
meNOG14780	243	9606.ENSP00000271707	39S ribosomal protein L9, mitochondrial precursor (L9mt)
meNOG14816	330	9606.ENSP00000351932	non supervised orthologous group
meNOG14864	247	9606.ENSP00000246912	
meNOG14866	194	9606.ENSP00000203363	
meNOG14887	311	9606.ENSP00000264228	Steroid 5 alpha-reductase
meNOG14894	316	9606.ENSP00000318966	Pin2-Interacting protein X1
meNOG14897	239	9606.ENSP00000261406	Probable ribosome biogenesis protein NEP1
meNOG14910	581	9606.ENSP00000289422	Pro-Neuregulin-2, membrane-bound isoform precursor (Pro-NRG2) Contains: Neuregulin-2
meNOG14934	247	9606.ENSP00000346412	non supervised orthologous group
meNOG15063	220	9606.ENSP00000282728	Homeobox protein PRH (Hematopoietically expressed homeobox)
meNOG15110	289	9606.ENSP00000279389	non supervised orthologous group
meNOG15115	219	9606.ENSP00000265881	
meNOG15116	213	9606.ENSP00000265882	RNA binding motif protein 7
meNOG15118	268	9606.ENSP00000259463	
meNOG15201	928	9606.ENSP00000339529	Ribonucleases P/MRP protein subunit POP1
meNOG15337	259	9606.ENSP00000300452	Protein involved in ubiquinone biosynthesis
meNOG15411	326	9606.ENSP00000352360	non supervised orthologous group
meNOG15450	481	9606.ENSP00000255108	
meNOG15704	236	9606.ENSP00000263657	
meNOG15732	198	9606.ENSP00000278483	non supervised orthologous group
meNOG15771	230	9606.ENSP00000245539	
meNOG15861	201	9606.ENSP00000306603	
meNOG15955	224	9606.ENSP00000257724	Myod family inhibitor domain containing protein
meNOG16120	257	9606.ENSP00000209779	non supervised orthologous group

meNOG16125	206	9606.ENSP00000226796	
meNOG16261	235	9606.ENSP00000310440	Charged multivesicular body protein 2a (Chromatin modifying protein 2a) Vacuolar protein sorting 2
meNOG16318	273	9606.ENSP00000317297	Aminomethyltransferase
meNOG16349	249	9606.ENSP00000215917	Srr1-Like protein
meNOG16399	281	9606.ENSP00000233713	
meNOG16464	208	9606.ENSP00000248342	Eukaryotic translation initiation factor 3 subunit
meNOG16643	226	9606.ENSP00000332287	Synaptogyrin 1 protein
meNOG16669	225	9606.ENSP00000343458	B-Cell receptor-associated protein 31 (BCR-associated protein Bap31)
meNOG16692	217	9606.ENSP00000354525	
meNOG16721	224	9606.ENSP00000308332	non supervised orthologous group
meNOG16779	237	9606.ENSP00000350144	Ischemia/Reperfusion inducible protein
meNOG16785	193	9606.ENSP00000246311	
meNOG16845	278	9606.ENSP00000346483	
meNOG16850	229	9606.ENSP00000221855	Cytoskeleton-Associated protein 1
meNOG16980	285	9606.ENSP00000309372	Yip1 interacting factor homolog
meNOG17051	301	9606.ENSP00000294119	
meNOG17085	211	9606.ENSP00000303145	Transmembrane trafficking protein
meNOG17090	244	9606.ENSP00000340798	Protein C1orf43
meNOG17105	234	9606.ENSP00000225609	N-Acetylglucosaminyl-Phosphatidylinositol de-N-acetylase
meNOG17163	217	9606.ENSP00000320508	Ring finger protein
meNOG17209	174	9606.ENSP00000296214	non supervised orthologous group
meNOG17247	216	9606.ENSP00000196551	Ribosomal protein S5
meNOG17325	181	9606.ENSP00000347836	Ubiquitin-Conjugating enzyme E2H
meNOG17331	179	9606.ENSP00000300087	
meNOG17350	181	9606.ENSP00000261636	
meNOG17356	231	9606.ENSP00000344942	Protein involved in nervous system development
meNOG17372	173	9606.ENSP00000279022	Myosin regulatory light chain 2, smooth muscle isoform (Myosin RLC) protein
meNOG17388	187	9606.ENSP00000225430	60S ribosomal protein
meNOG17397	172	9606.ENSP00000254105	Rna-Binding protein 8A
meNOG17408	353	9606.ENSP00000353698	non supervised orthologous group
meNOG17410	198	9606.ENSP00000262225	Coated vesicle membrane protein
meNOG17435	206	9606.ENSP00000329686	A-Type potassium channel modulatory protein 1
meNOG17463	199	9606.ENSP00000223369	Prenylated SNARE protein
meNOG17471	175	9606.ENSP00000335636	
meNOG17474	186	9606.ENSP00000245304	Protein involved in small GTPase mediated signal transduction
meNOG17484	222	9606.ENSP00000299335	Cytochrome c oxidase assembly protein COX11, mitochondrial
meNOG17492	179	9606.ENSP00000270799	Ribosomal protein L11
meNOG17496	194	9606.ENSP00000270634	Ribosomal protein L13a; 60S ribosomal protein L13a; 23 kD highly basic protein
meNOG17500	186	9606.ENSP00000355102	60S ribosomal protein L17 (L23)
meNOG17501	204	9606.ENSP00000318995	Nadh-Ubiquinone oxidoreductase 20 kDa subunit, mitochondrial precursor
meNOG17539	232	9606.ENSP00000326254	non supervised orthologous group
meNOG17563	222	9606.ENSP00000275605	Phosphoserine phosphatase (EC 3.1.3.3) (PSP)
meNOG17569	195	9606.ENSP00000330884	non supervised orthologous group
meNOG17575	191	9606.ENSP00000330879	Ribosomal protein L9
meNOG17579	215	9606.ENSP00000262801	
meNOG17605	184	9606.ENSP00000310071	Anaphase promoting complex subunit 10 (APC10)
meNOG17606	177	9606.ENSP00000254940	60S ribosome subunit biogenesis protein NIP7 homolog
meNOG17620	302	9606.ENSP00000258603	non supervised orthologous group
meNOG17633	178	9606.ENSP00000236164	
meNOG17640	179	9606.ENSP00000295702	Translocon-Associated protein subunit beta precursor (TRAP-beta)
meNOG17666	192	9606.ENSP00000333833	Chemokine-Like factor
meNOG17680	202	9606.ENSP00000295899	non supervised orthologous group
meNOG17695	194	9606.ENSP00000296509	Mitotic spindle assembly checkpoint protein MAD2A (MAD2-like 1)
meNOG17696	252	9606.ENSP00000298406	Protein involved in valine, leucine and isoleucine degradation
meNOG17714	213	9606.ENSP00000279993	Protein involved in DNA alkylation
meNOG17727	237	9606.ENSP00000264993	Carnitine deficiency-associated gene expressed in ventricle protein

meNOG17730	178	9606.ENSP00000321571	
meNOG17763	176	9606.ENSP00000053468	Mitochondrial 28S ribosomal protein S10 (S10mt)
meNOG17773	175	9606.ENSP00000306548	39S ribosomal protein L13, mitochondrial (L13mt)
meNOG17861	195	9606.ENSP00000320236	Mitochondrial import inner membrane translocase subunit Tim22
meNOG17866	173	9606.ENSP00000318650	Cysteine knot superfamily protein
meNOG17874	186	9606.ENSP00000274458	
meNOG17928	148	9606.ENSP00000341170	PTN (Heparin-binding growth-associated molecule) Heparin-binding
meNOG17939	174	9606.ENSP00000258258	Probable UPF0334 kinase-like protein
meNOG17977	194	9606.ENSP00000211302	28S ribosomal protein S18a, mitochondrial precursor (MRP-S18-a)
meNOG18000	172	9606.ENSP00000329553	IMP2 inner mitochondrial membrane protein
meNOG18073	202	9606.ENSP00000280330	Methyltransferase
meNOG18099	166	9606.ENSP00000264871	Eukaryotic translation elongation factor 1 epsilon 1
meNOG18108	188	9606.ENSP00000084795	Ribosomal protein
meNOG18136	188	9606.ENSP00000332177	non supervised orthologous group
meNOG18165	173	9606.ENSP00000267750	non supervised orthologous group
meNOG18256	299	9606.ENSP00000285814	Mki67 (FHA domain) interacting nucleolar phosphoprotein
meNOG18282	210	9606.ENSP00000261817	
meNOG18295	165	9606.ENSP00000316787	non supervised orthologous group
meNOG18298	242	9606.ENSP00000184772	non supervised orthologous group
meNOG18335	167	9606.ENSP00000263235	Mitochondrial import inner membrane translocase subunit Tim17 B (JM3)
meNOG18407	135	9606.ENSP00000263270	Adaptor-Related protein complex 2, sigma 1 subunit
meNOG18478	141	9606.ENSP00000269601	Protein involved in mitosis
meNOG18486	138	9606.ENSP00000222969	
meNOG18509	167	9606.ENSP00000310360	non supervised orthologous group
meNOG18531	149	9606.ENSP00000211372	Ribosomal protein S18
meNOG18575	230	9606.ENSP00000341504	ATP binding protein
meNOG18582	145	9606.ENSP00000346015	Ribosomal protein L27a
meNOG18588	151	9606.ENSP00000270625	Ribosomal protein S11
meNOG18600	143	9606.ENSP00000296674	Ribosomal protein
meNOG18616	166	9606.ENSP00000286175	Isomerase-Like
meNOG18633	146	9606.ENSP00000311028	40S ribosomal protein
meNOG18656	157	9606.ENSP00000276689	Protein involved in mitochondrial electron transport, NADH to ubiquinone
meNOG18688	150	9606.ENSP00000251453	40S ribosomal protein S16
meNOG18707	145	9606.ENSP00000233609	Ribosomal protein S15
meNOG18711	161	9606.ENSP00000253686	Mitochondrial 28S ribosomal protein S25 (S25mt)
meNOG18726	169	9606.ENSP00000294360	non supervised orthologous group
meNOG18742	212	9606.ENSP00000261758	Mesoderm development candidate 2 protein
meNOG18807	131	9606.ENSP00000263495	MAPK activating protein PM20,PM21
meNOG18811	174	9606.ENSP00000260443	Probable ribosome biogenesis protein
meNOG18815	153	9606.ENSP00000299761	non supervised orthologous group
meNOG18820	151	9606.ENSP00000228140	Ribosomal protein S13
meNOG18936	209	9606.ENSP00000312599	non supervised orthologous group
meNOG18954	164	9606.ENSP00000282185	Protein involved in protein modification
meNOG18969	174	9606.ENSP00000288937	Mitochondrial ribosomal protein L17
meNOG18996	152	9606.ENSP00000243045	non supervised orthologous group
meNOG19001	165	9606.ENSP00000253452	Cytochrome c oxidase subunit IV isoform 1, mitochondrial precursor (EC 1.9.3.1)
meNOG19046	183	9606.ENSP00000355203	Mitochondrial ribosomal protein L21
meNOG19067	157	9606.ENSP00000317121	non supervised orthologous group
meNOG19079	163	9606.ENSP00000354590	non supervised orthologous group
meNOG19086	190	9606.ENSP00000238628	non supervised orthologous group
meNOG19088	154	9606.ENSP00000216416	Protein involved in signal transduction
meNOG19142	201	9606.ENSP00000316262	non supervised orthologous group
meNOG19143	143	9606.ENSP00000229903	non supervised orthologous group
meNOG19211	178	9606.ENSP00000333666	Protein involved in 1- and 2-Methylnaphthalene degradation
meNOG19225	154	9606.ENSP00000350098	Processing of precursor 5, ribonuclease P/MRP family
meNOG19251	281	9606.ENSP00000288063	Mrna transport regulator 3 protein
meNOG19283	141	9606.ENSP00000346039	60S ribosomal protein L23

meNOG19337	144	9606.ENSP00000354426	15 kDa selenoprotein
meNOG19442	232	9606.ENSP00000227520	non supervised orthologous group
meNOG19495	131	9606.ENSP00000318646	40S ribosomal protein
meNOG19501	172	9606.ENSP00000352744	Ribosomal protein L12
meNOG19537	128	9606.ENSP00000341176	Ribosomal protein
meNOG19629	180	9606.ENSP00000248114	Growth factor, erv1 (S. cerevisiae)-like
meNOG19651	172	9606.ENSP00000349801	non supervised orthologous group
meNOG19667	140	9606.ENSP00000350378	non supervised orthologous group
meNOG19700	141	9606.ENSP00000282892	Protein involved in transcription from RNA polymerase II promoter
meNOG19746	129	9606.ENSP00000215960	Dna-Directed RNA polymerases I, II, and III 14.4 kDa polypeptide (EC 2.7.7.6)
meNOG19762	134	9606.ENSP00000255764	DNA segment Chr 13 Wayne State University 50 expressed protein
meNOG19765	139	9606.ENSP00000265304	Single-Stranded DNA-binding protein, mitochondrial precursor (Mt-SSB)
meNOG19802	183	9606.ENSP00000296389	non supervised orthologous group
meNOG19850	148	9606.ENSP00000262428	Coactosin-Like protein
meNOG19889	118	9606.ENSP00000227618	non supervised orthologous group
meNOG19890	125	9606.ENSP00000226522	Mitogen-Activated protein kinase kinase 1 interacting protein 1; MEK
meNOG19895	124	9606.ENSP00000259469	60S ribosomal protein L35
meNOG19906	135	9606.ENSP00000284000	Ccaat/Enhancer-Binding protein gamma (C/EBP gamma)
meNOG19921	126	9606.ENSP00000217103	Prefoldin subunit 4
meNOG19942	129	9606.ENSP00000314625	Leptin receptor overlapping transcript-like 1
meNOG19950	135	9606.ENSP00000273986	non supervised orthologous group
meNOG19970	158	9606.ENSP00000260270	Adrenodoxin, mitochondrial precursor (Adrenal ferredoxin)
meNOG19973	153	9606.ENSP00000216034	Mitochondrial import receptor subunit TOM22 homolog (Translocase of outer membrane 22 kDa subunit homolog)
meNOG19977	113	9606.ENSP00000273480	Ring-Box protein 2 (Rbx2) (RING finger protein 7)
meNOG19980	117	9606.ENSP00000222547	BET1 homolog (Golgi vesicular membrane trafficking protein p18)
meNOG20004	127	9606.ENSP00000239463	Mitochondrial 28S ribosomal protein S14
meNOG20013	148	9606.ENSP00000271485	Prefoldin subunit 2
meNOG20063	140	9606.ENSP00000293777	non supervised orthologous group
meNOG20090	178	9606.ENSP00000309376	Protein involved in heterophilic cell adhesion
meNOG20114	120	9606.ENSP00000316222	Glycoprotein hormone beta 5 precursor (Thyrostimulin beta subunit)
meNOG20175	139	9606.ENSP00000310935	Fk506-Binding protein 2
meNOG20212	179	9606.ENSP00000265677	39S ribosomal protein L18, mitochondrial precursor
meNOG20340	127	9606.ENSP00000202017	P53 and DNA damage-regulated protein
meNOG20442	124	9606.ENSP00000215565	NADH dehydrogenase (ubiquinone) 1 beta subcomplex, 7
meNOG20617	157	9606.ENSP00000265264	60S ribosomal protein
meNOG20653	131	9606.ENSP00000225728	Mediator of RNA polymerase II transcription subunit 31 (Mediator complex subunit SOH1)
meNOG20762	129	9606.ENSP00000221784	
meNOG20806	94	9606.ENSP00000266735	Small nuclear ribonucleoprotein F (snRNP-F)
meNOG20812	124	9606.ENSP00000315693	Mitochondria-Associated granulocyte macrophage CSF signaling molecule
meNOG20831	123	9606.ENSP00000250101	Thioredoxin-Like 5 protein
meNOG20864	120	9606.ENSP00000267884	Signal recognition particle 14 kDa protein
meNOG20953	114	9606.ENSP00000256644	Hepatitis B virus x-interacting protein; HBx-interacting protein
meNOG20974	96	9606.ENSP00000300461	non supervised orthologous group
meNOG21010	88	9606.ENSP00000223084	LSM5 homolog, U6 small nuclear RNA associated protein
meNOG21047	94	9606.ENSP00000334960	non supervised orthologous group
meNOG21169	82	9606.ENSP00000280354	Mitochondrial import inner membrane translocase subunit Tim8 B
meNOG21214	97	9606.ENSP00000327467	non supervised orthologous group
meNOG21336	103	9606.ENSP00000281031	NADH dehydrogenase (ubiquinone)
meNOG21590	153	9606.ENSP00000298818	HESB like domain containing 1 protein
meNOG21830	72	9606.ENSP00000309619	non supervised orthologous group
meNOG22133	98	9606.ENSP00000254616	Mitochondrial import inner membrane translocase subunit TIM9 B (Fracture callus protein 1)
