# Supplementary material for: Identifying Single Copy Orthologs in Metazoa
Source: PLoS Comput Biol. 2011 Dec 1;7(12):e1002269. doi: 10.1371/journal.pcbi.1002269 (PMC3228760; doi:10.1371/journal.pcbi.1002269)
Supplement: Table S4 — Details of the draft or recently published genomes assessed. NCBI = National Center for Biotechnology Information. JGI = Joint Genome Institute (These sequence data were produced by the US Department of Energy Joint Genome Institute http://www.jgi.doe.gov/ in collaboration with the user community). (PDF) [file pcbi.1002269.s011.pdf]

TaxID	Name	#Total Genes	Genome publication or release(*) date	Number of single copy orthologs identified	% of total single copy orthologs	Version
81824	*Monosiga brevicollis*	9,196	14/02/2008	776	69	JGI v1.0
73382	*Capitella sp.*	32,415	20/09/2007*	1052	93	JGI v1.0
10228	*Trichoplax adhaerens*	11,520	21/08/2008	933	83	JGI v1.0
7739	*Branchiostoma floridae*	50,817	19/06/2008	1084	96	JGI v1.0
6412	*Helobdella robusta*	23,432	23/07/2007*	983	87	JGI v1.0
45351	*Nematostella vectensis*	27,273	06/07/2007	1025	91	JGI v1.0
7668	*Strongylocentrotus purpuratus*	29,128	10/11/2006	1057	94	NCBI Spur_v2.1
