# Supplementary material for: Identifying Single Copy Orthologs in Metazoa
Source: PLoS Comput Biol. 2011 Dec 1;7(12):e1002269. doi: 10.1371/journal.pcbi.1002269 (PMC3228760; doi:10.1371/journal.pcbi.1002269)
Supplement: Table S5 — EST datasets assessed for completeness. The EST datasets assessed for completeness as part of this study. (PDF) [file pcbi.1002269.s012.pdf]

TaxID	Name	Number of ESTs in dataset	Number of single copy genes	% of total single copy orthologs
6085	*Hydra magnipapillata*	164,325	646	57
6161	*Dugesia japonica*	7,666	174	15
6210	*Echinococcus granulosus*	10,003	147	13
6221	*Cerebratulus lacteus*	6,317	85	8
6431	*Urechis caupo*	2,208	60	5
6526	*Biomphalaria glabrata*	158,327	287	25
6565	*Crassostrea virginica*	14,652	280	25
6613	*Euprymna scolopes*	42,468	33	3
6759	*Carcinus maenas*	24,569	484	43
6848	*Carcinoscorpius rotundicauda*	579	20	2
6941	*Rhipicephalus microplus*	52,747	626	56
7594	*Patiria pectinifera*	56,854	307	27
7757	*Petromyzon marinus*	120,731	587	52
7764	*Eptatretus burgeri*	23,886	372	33
10195	*Brachionus plicatilis*	3,552	73	6
10212	*Bugula neritina*	3,360	51	5
10224	*Saccoglossus kowalevskii*	202,190	844	75
27923	*Mnemiopsis leidyi*	15,752	228	20
29022	*Scutigera coleoptrata*	2,400	44	4
29158	*Mytilus galloprovincialis*	14,760	248	22
31199	*Argopecten irradians*	7,700	77	7
34513	*Terebratalia transversa*	3,552	125	11
34521	*Phoronis ijimai*	2,208	11	1
35525	*Daphnia magna*	13,270	178	16
35630	*Hydractinia echinata*	9,464	145	13
35632	*Lumbricus rubellus*	20,136	201	18
37621	*Priapulus caudatus*	2,306	69	6
43123	*Pedicellina cernua*	5,184	66	6
45264	*Acropora millepora*	10,247	325	29
46003	*Xiphinema index*	9,351	273	24
52888	*Spadella cephaloptera*	11,939	62	6
55567	*Suberites domuncula*	299	3	0
58794	*Chaetopleura apiculata*	2,304	17	2
63121	*Ptychodera flava*	3,360	58	5
66402	*Neochildia fusca*	1,728	15	1
67896	*Cristatella mucedo*	3,264	67	6
72734	*Reniera*	83,040	459	41
79327	*Schmidtea mediterranea*	74,915	656	58

81526	*Monosiga ovata*	76,534	384	34
84072	*Symsagittifera roscoffensis*	846	14	1
96448	*Philodina roseola*	3,168	86	8
115339	*Acanthoscurria gomesiana*	6,790	250	22
139456	*Fenneropenaeus chinensis*	10,506	91	8
140457	*Pleurobrachia pileus*	8,540	354	31
187807	*Carinoma mutabilis*	3,168	50	4
190581	*Spinochordodes tellinii*	2,208	25	2
216211	*Themiste lageniformis*	2,640	61	5
225164	*Lottia gigantea*	252,091	973	86
232323	*Hypsibius dujardini*	5,235	113	10
242395	*Xenoturbella bocki*	3,840	67	6
245088	*Richtersius coronifer*	3,360	86	8
256131	*Chaetoderma nitidulum*	1,631	26	2
279730	*Haementeria depressa*	891	39	3
282301	*Macrostomum lignano*	7,617	152	13
338297	*Gnathostomula peregrina*	3,552	63	6
386100	*Oscarella carmela*	11,176	180	16
447489	*Myzostoma seymourcollegiorum*	1,056	43	4
488521	*Anoplodactylus eroticus*	3,744	76	7
488522	*Echinoderes horni*	3,264	77	7
488523	*Euperipatoides kanangrensis*	3,360	72	6
488524	*Turbanella ambronensis*	3,264	28	2
488525	*Paraplanocera*	3,744	55	5
