# Supplementary material for: Identifying Single Copy Orthologs in Metazoa
Source: PLoS Comput Biol. 2011 Dec 1;7(12):e1002269. doi: 10.1371/journal.pcbi.1002269 (PMC3228760; doi:10.1371/journal.pcbi.1002269)
Supplement: Table S6 — Distribution of single copy meNOGs according to species composition. The distribution of single copy meNOGs according to species composition. (PDF) [file pcbi.1002269.s013.pdf]

Number of species	Number of single copy meNOG
2	2,815
3	2,180
4	792
5	640
6	660
7	773
8	766
9	702
10	766
11	853
12	977
13	780
14	482
15	489
16	536
17	478
18	219
